# Cornelian Cherry (*Cornus mas* L.) Extracts Exert Cytotoxicity in Two Selected Melanoma Cell Lines—A Factorial Analysis of Time-Dependent Alterations in Values Obtained with SRB and MTT Assays

**DOI:** 10.3390/molecules27134193

**Published:** 2022-06-29

**Authors:** Łukasz Lewandowski, Iwona Bednarz-Misa, Alicja Z. Kucharska, Agnieszka Kubiak, Patrycja Kasprzyk, Tomasz Sozański, Dominika Przybylska, Narcyz Piórecki, Małgorzata Krzystek-Korpacka

**Affiliations:** 1 Department of Medical Biochemistry, Wroclaw Medical University, Chalubinskiego 10, 50-368 Wroclaw, Poland; iwona.bednarz-misa@umw.edu.pl (I.B.-M.); agnieszka.kubiak70@gmail.com (A.K.); patrycja.kasprzyk@umw.edu.pl (P.K.); malgorzata.krzystek-korpacka@umw.edu.pl (M.K.-K.); 2 Department of Fruit, Vegetable and Plant Nutraceutical Technology, Wroclaw University of Environmental and Life Sciences, J. Chelmonskiego 37, 51-630 Wroclaw, Poland; dominika.przybylska@upwr.edu.pl; 3 Department of Pharmacology, Wroclaw Medical University, J. Mikulicza-Radeckiego 2, 50-345 Wroclaw, Poland; tomasz.sozanski@umw.edu.pl; 4 Bolestraszyce Arboretum and Institute of Physiography, Bolestraszyce 130, 37-722 Wyszatyce, Poland; npiorecki@ur.edu.pl; 5 Institute of Physical Culture Sciences, Medical College, University of Rzeszow, Cicha 2A, 35-326 Rzeszow, Poland

**Keywords:** A375 cell line, cell culture, cell viability, contrast analysis, cornelian cherry, *Cornus mas* L., cytotoxicity, melanoma, MeWo cell line

## Abstract

Despite the fact that phytochemicals of *Cornaceae* species have long been discussed as possible auxiliary agents in contemporary treatment, the insights on their properties remain relatively scarce. This study focuses on *Cornus mas* L. (Cornelian cherry), the extracts of which are reported to exert a pleiotropic effect shown in both in vivo and in vitro studies. This study aimed to explore the cytotoxic effect of extracts from fruits of red (*Cornus mas* L. ‘Podolski’) and yellow (*Cornus mas* L. ‘Yantarnyi’ and ‘Flava’) Cornelian cherries on two melanoma cell lines (A375 and MeWo). The extracts were characterized in the context of the concentration of bioactive compounds of antioxidative properties. Cytotoxicity was investigated with the use of the following two assays: SRB and MTT. An additional, alternative protocol for the SRB assay was used in this study so as to account for possible bias. Cytotoxicity was assessed as a difference in the whole time series of cell viability, instead of analyzing differences in raw values (often found in the literature). Both extracts from *Cornus mas* L. induced cytotoxicity in both A375 and MeWo cell lines, although the response of these cells was different. Moreover, based on this study, there is no evidence for claiming a different magnitude of cytotoxicity between these two extracts.

## 1. Introduction

The notoriety of melanoma stems from its high phenotype plasticity, which does not only increase the probability of the metastasis of this tumor (compared to other skin cancers) but also enables melanoma cells to rapidly adjust their transcriptional profile to the alterations within the tumor microenvironment, associated with the presence of various non-cancer cells and/or presence of different compounds, including drugs [1,2,3,4,5,6]. This ability renders melanoma cells more resistant to targeted therapy and immunotherapy [5,6,7,8]. The introduction of phytochemicals as a potentially auxiliary factor in the antitumor treatment of melanoma is lately being discussed in the literature since many plant-derived compounds (in the following various forms: as plant extracts, single isolated compounds or compounds transported with nanocarriers) have yielded promising results against epithelial-mesenchymal transition, survival, invasion and metastatic capabilities of melanoma cells [9,10,11,12,13,14,15,16,17,18,19,20,21].

Due to their broad spectrum of utility, *Cornaceae* have long been discussed as a family of potential auxiliary uses in medicine, the food industry and cosmetics manufacturing. The scientific database concerning one of the major representants of this family, the ‘Cornelian cherry’ (*Cornus mas* L.), has reached over 4800 records. Such interest in this species stems from the medical property of compounds [22,23,24] (mainly—flavonoids, anthocyanins and iridoids) found in both the following: its leaves and fruits [25,26,27]. According to the literature, extracts from *C. mas* L. possess antibacterial [28,29,30,31,32] and antifungal [33] activity. Moreover, anti-inflammatory [34,35] and antioxidative [34,35,36,37,38] properties of *C. mas* L. extracts (and fruit preserves [39]) may explain hepatoprotective [40,41,42], cardioprotective [43,44], nephroprotective [45,46], anti-atherosclerotic [47,48,49], antidiabetic [50], hypoglycemic and hypocholesterolemic [51,52,53,54,55] effects of *C. mas* L. observed in animal models.

Much attention has been drawn to the cytotoxic, antiproliferative, and thus, anti-cancer [38,56,57,58,59,60,61] attributes of *C. mas* L. Furthermore, the antitumor and anti-inflammatory actions of *C. mas* L. compounds have been successfully applied in the form of nanoparticle carriers containing the extract itself or its various components [9,62,63,64,65,66]. Cytotoxic/antiproliferative properties of *C. mas* L. extracts have been observed (based on the aforementioned studies) with the use of various tumor cell lines, such as the following: MCF-7, SKOV-3, PC-3, HeLa, HepG2, CaCo-2, HT29, CT26, A549. However, although some studies suggest that an extract from the fruits of *C. officinalis* L. inhibits the advanced glycation end-product-induced melanogenesis process in melanoma (B16 cell line) cells [67], no information on the cytotoxic effect of *C. mas* L. extracts on melanoma cell lines could be found in the literature. This study aimed to explore the possible cytotoxic effect of two types (yellow and red) of *C. mas* L. extracts on the following two melanoma cell lines of different growth rates: A375 and MeWo.

## 2. Results

### 2.1. The Chemical Composition of Cornelian Cherry Extracts

**Figure 1:**
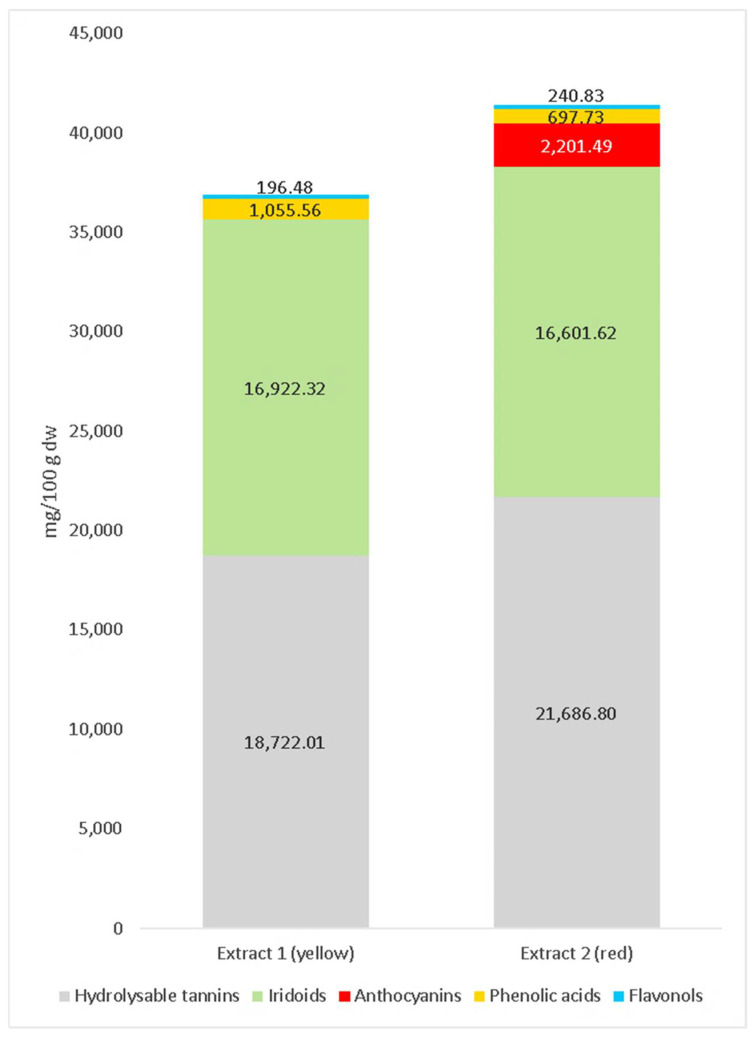
Content (mg/100 g dry weight (dw)) of main groups compounds of extracts from yellow and red Cornelian cherry (*Cornus mas* L.) fruits identified by means of HPLC method.

The quantitative results concerning selected iridoids, anthocyanins, phenolic acids, flavonols and hydrolyzable tannins of Cornelian cherry extracts used in this study are shown in Supplementary Materials Table S1 and Figure 1. The compounds were identified based on their elution order, retention times, spectra of the individual peaks (MS, MS/MS); additionally, by comparison with literature data [24,32,50,68]. The study resulted in the identification of the following 37 main compounds: 2 iridoids (loganic acid and cornuside with pseudomolecular ions [M − H]^−^ at *m*/*z* 375 and 541), 4 anthocyanins (cyanidin 3-*O*-galactoside, cyanidin 3-*O*-robinobioside, pelargonidin 3-*O*-galactoside and pelargonidin 3-*O*-robinobioside with [M + H]^+^ at *m*/*z* 449, 595, 433 and 579 respectively), 3 phenolic acids (caftaric acid and coutaric acid with [M − H]^−^ at *m*/*z* 311 and 295, respectively), 2 flavonols (quercetin 3-O-glucuronide and kaempferol 3-*O*-galactoside with [M − H]^−^ at *m*/*z* 477 and 447, respectively) and 26 hydrolyzable tannins, including their spatial isomers. Among hydrolyzable tannins, the main compounds were gemin D—the simplest molecule of all ellagitannins with ion [M − H]^−^ at *m*/*z* 633 and its two derivatives (tellimagrandin I with [M − H]^−^ at *m*/*z* 785 and tellimagrandin II with [M − H]^−^ at *m*/*z* 937), two dimeric ellagitannins (camptothin A, which produced two ions [M − 2H]^–2^ at *m*/*z* 708 and [M − H]^–^ at *m*/*z* 1417 and cornusiin A with two ions, [M − 2H]^–2^ at *m*/*z* 784 and [M − H]^–^ at *m*/*z* 1569) and two trimeric ellagitannins (cornusiin F, which produced two ions, [M − 2H]^–2^ at *m*/*z* 1100 and [M − H]^–^ at *m*/*z* 2201 and cornusiin C, which produced two ions, [M − 2H]^–2^ at *m*/*z* 1176 and [M − H]^–^ at *m*/*z* 2353). Among the identified phenolic compounds, coutaric acid and hydrolyzable tannins were identified in the extracts of Cornelian cherry (*Cornus mas* L.) fruit for the first time. In previous studies, tannins were determined in Cornelian cherry but only in leaf and stone, not in fruit [29,68]. The contents of compounds of extracts are shown in Table 1.

The extract from the yellow fruits did not contain anthocyanins and was composed mainly of iridoids, hydrolyzable tannins and a small number of phenolic acids and flavonols. The content of loganic acid was in the amount of 15,383.35 mg/100 g dry weight (dw). Three phenolic acids present in the extract constituted only 1055.56 mg/100 g dw while flavonols 196.48 mg/100 g dw. The content of hydrolyzable tannins was in the amount of 18,722.01 mg/100 g dw.

The extract from the red fruits of the Cornelian cherry abounded in most of the identified compounds. It contained 16,601.62 mg/100 g dw iridoids, 2201.49 mg/100 g dw anthocyanins, 697.73 mg/100 g dw phenolic acids, 240.83 mg/100 g dw flavonols and 21,686.80 mg/100 g dw hydrolyzable tannins. The quantitative and qualitative composition of the iridoids and phenolic compounds of both extracts is comparable, as described by Dzydzan et al. [50].

### 2.2. Measuring Cytotoxicity with Use of SRB and MTT Methods

As mentioned before, the data presented in this section refer to two measurement procedures. The ‘standard procedure’ was carried out according to standard SRB method guidelines—trichloroacetic acid was added directly to the culture medium after reaching the end of the appropriate growth period (6 h, 24 h, 48 h, 72 h). The ‘alternative procedure’ involved removing the culture medium before adding trichloroacetic acid. In that case, the acid was diluted to reflect the conditions followed in the standard procedure. The rationale behind the analysis of an additional procedure is the suspected impact of the presence of Cornelian cherry extracts (*per se*) in the culture medium on the obtained results—due to the additional protein content found in these extracts.

Such an additional procedure was unnecessary in the context of the MTT method, as the removal of culture medium before further measurement steps was a part of the standard assay protocol since Cornelian cherry extracts possess antioxidative potential.

The report from the analysis of variance for all of the results is given in Table 1. A map of *p*-values for the contrast analysis is shown in Table 2. Due to the vast amount of data regarding the descriptive statistics of each discussed interaction, the tables which show marginal values (associated with the figures in this section) are given in Appendix A (Table A2, Table A3 and Table A4). In the whole ‘Results’ section, the results are described in reference to α-value of 0.05.

#### 2.2.1. The Series Measured with the SRB Method

**Figure 2:**
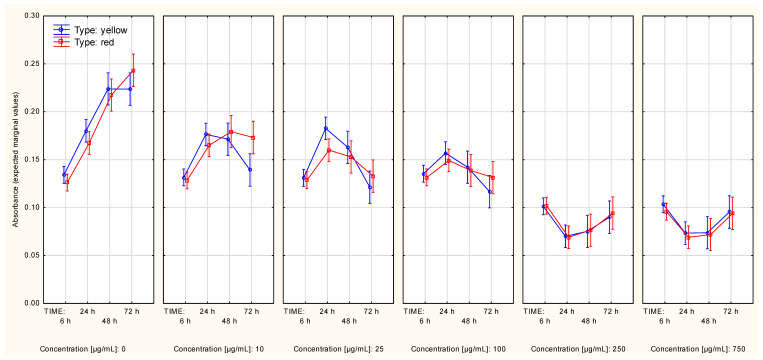
Cell protein content curves (A375 cell line, SRB assay) in context of both: type and concentration of Cornelian cherry extracts (Time*Type*Concentration interaction). The values were obtained with use of the alternative assay protocol. The values are given as estimated marginal means ± standard error.

Under no presence of Cornelian cherry extracts, the cell protein content of A375 cells reached a plateau approximately at the 48th to 72nd hour, regardless of the assay procedure. The alternative SRB procedure showed significant differences in cell quantity over time in the context of extract type (Figure S1A) or concentration (Figure S1B). However, the difference between the influence of these extracts on cell protein content was on the brink of statistical significance (approximately, *p* = 0.062) when the growth curves were split according to extract concentration (Figure 2).

The statistical significance of the difference between cell protein content curves in the context of different extract types was affected by the higher slope of the growth curve in the 6–24 h time period and a negative slope in the 48 h–72 h time period, which was obtained for measurement series associated with the presence of the extract from yellow Cornelian cherry. Under the presence of an increasing concentration of extracts, the cell count limit was decreasing, reaching a value close to “0” in the following two highest concentrations of Cornelian cherry extracts: 250 µg/mL and 750 µg/mL (Figure S1B). Contrast analysis revealed significant differences between the control series (concentration equal to “0”) and the other series, starting from the following lowest concentration tested: 10 µg/mL (Table 2).

**Figure 3:**
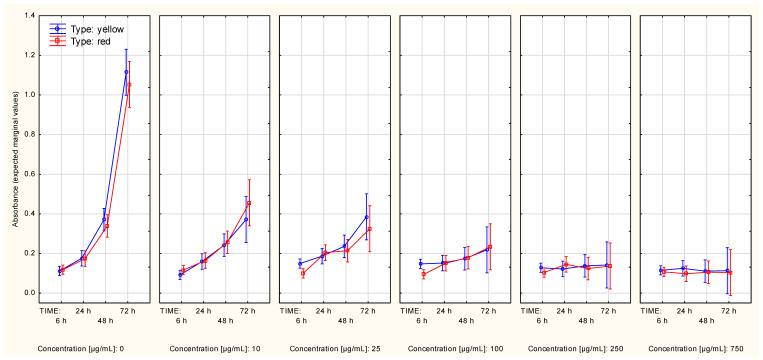
Cell protein content curves (A375 cell line, SRB assay) in context of both: type and concentration of Cornelian cherry extracts (Time*Type*Concentration interaction). The values were obtained with use of the standard assay protocol. The values are given as estimated marginal means ± standard error.

The standard assay procedure revealed no difference in cell protein content curves in the context of the type of the used extract (Figure S2A). The growth of the cells was markedly decreasing with increasing values of extract concentration. No growth was observed in the following two highest concentrations: 250 µg/mL and 750 µg/mL (Figure S2B). When the curves were split, simultaneously, according to both extract type and concentration, the two types of extracts showed no difference in how they affected the changes in cell protein content (Figure 3). Contrast analysis confirmed the observations made with the use of the standard assay procedure—a significant difference in growth curves, compared to the control series, was found in all of the analyzed series (starting from a concentration of *Cornus mas* L. extract equal to 10 µg/mL).

**Figure 4:**
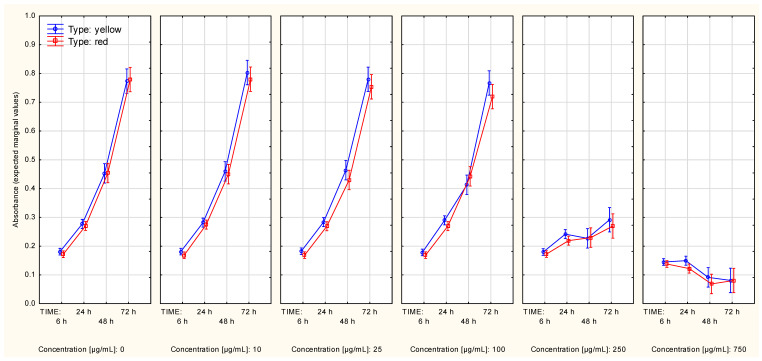
Cell protein content curves (MeWo cell line, SRB assay) in context of both: type and concentration of Cornelian cherry extracts (Time*Type*Concentration interaction). The values were obtained with use of the alternative assay protocol. The values are given as estimated marginal means ± standard error.

The cell protein content plateau of the MeWo cells was not reached in the control series regardless of the used assay procedure. The alternative procedure revealed that the difference in extract type did not have a significant influence over cell protein content alterations (Figure S3A), regardless of whether the data was additionally split according to extract concentration (Figure 4). Although the two highest concentrations (250 µg/mL and 750 µg/mL) highly affected changes in cell protein content, contrast analysis revealed a slight difference (in the growth interval from 24th up to 72nd hour of growth) between the control series and the series in which the concentration was 100 µg/mL, regardless of the type of extract (Table 2, Figure S3B).

**Figure 5:**
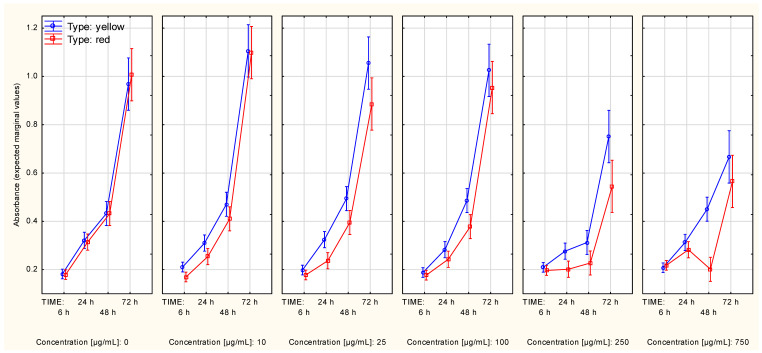
Cell protein content curves (MeWo cell line, SRB assay) in context of both: type and concentration of Cornelian cherry extracts (Time*Type*Concentration interaction). The values were obtained with use of the standard assay protocol. The values are given as estimated marginal means ± standard error.

Interestingly, the standard assay procedure showed differences in alterations in cell protein content slopes of the MeWo cells between series associated with a different type of the extract (Figure S4A). The series associated with an extract concentration equal to 10 µg/mL showed slightly increased cell protein content in comparison to the control series (Figure 5). These two occurrences may be associated with the significance of the Time*Type*Concentration interaction (Table 1). Contrast analysis revealed that the differences in the cell protein content trend occurred in the two highest extract concentrations, regardless of the extract type (Table 2; this fact could also be seen in Figure S4B).

#### 2.2.2. Measurements of Cell Metabolic Activity with Use of the MTT Method

Regarding the control series, conversely to the observations for the SRB method, no plateau was reached in the case of A375 cells. MeWo cells reached their metabolic capacity plateau approximately at the 48th/72nd hour of growth.

**Figure 6:**
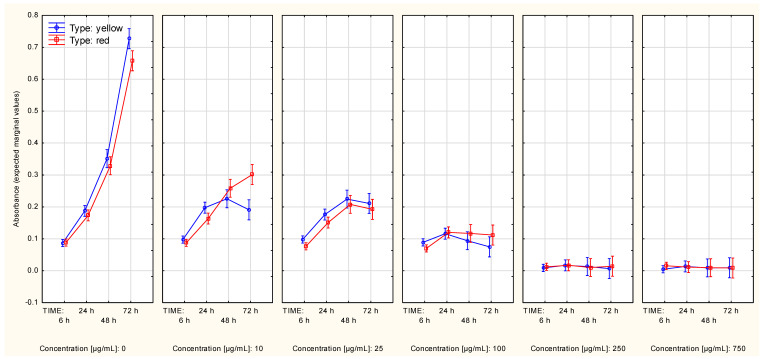
Metabolic activity curves (A375 cell line, MTT assay) in context of both: type and concentration of Cornelian cherry extracts (Time*Type*Concentration interaction). The values are given as estimated marginal means ± standard error.

In the context of the A375 cells, the between-extract type differences in the first two time points (6 h, 24 h) most probably were associated with the significance of the Time*Type interaction (Figure S5A). After splitting the data according to both the following: type and concentration of the extract, the difference between metabolic activity curves associated with the two extract types was observed in the data associated with an extract concentration of 10 µg/mL (Figure 6)—thus, the significance of the Time*Type*Concentration interaction (Table 1). Contrast analysis showed significant differences in the overall metabolic activity curve between the control series and the rest of the series, starting from the lowest tested extract concentration (10 µg/mL), regardless of extract type. This dependence could also be seen in the metabolic activity curves if extract type was not accounted for (Figure S5B). The two highest extract concentrations were associated with very low cell metabolic activity, which was maintained over the analyzed time.

**Figure 7:**
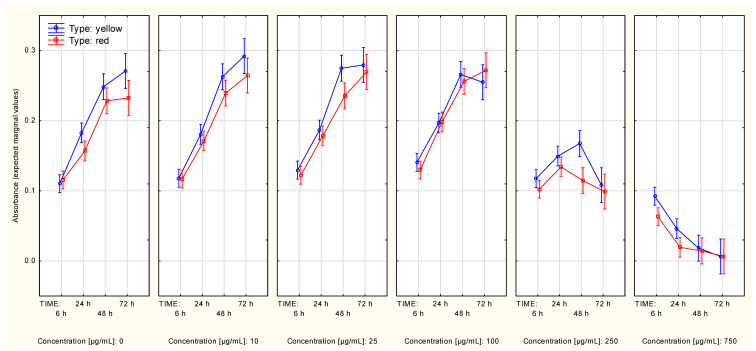
Metabolic activity curves (MeWo cell line, MTT assay) in context of both: type and concentration of Cornelian cherry extracts (Time*Type*Concentration interaction. The values are given as estimated marginal means ± standard error.

Significant differences in two sets of series measured in the context of the MeWo cells, associated with different extract types (Figure S6A), were observed. The differences in cell growth remained significant when both the following factors: extract type and concentration, were accounted for (Figure 7). When the extract type was not accounted for, the two highest extract concentrations (250 µg/mL and 750 µg/mL) were associated with different metabolic activity curves, compared to the control series (Figure S6B). The results of contrast analysis reflected the differences in metabolic activity seen in Figure S6A, showing variable results depending on extract type. The lowest concentration of the yellow extract, which had a significant impact on cell metabolic activity, was 100 µg/mL. The red extract, however, showed a significant impact on cell metabolic activity only when the first time point (6 h) was compared with the other three time points (24 h, 48 h, 72 h). Overall, both extract types, in a concentration of 250 µg/mL or 750 µg/mL, had an impact on cell metabolic activity over time.

### 2.3. Estimation of IC_50_ Based on the Results from SRB and MTT Assays

In the previous sections, cytotoxicity was assessed as the difference in the shape of the curve describing the changes in cell viability over time. Whereas that reasoning allowed the use of more sensitive statistical methods to test whether the growth rates differed under the effect of *C. mas* L. extracts, it may seem confusing in the context of describing the cytotoxicity in the context of IC_50_. Therefore, the data in this section have been transformed from raw absorbance values to a percentage of cell viability (in reference to the control values). The data is shown in a series describing cell viability in different concentrations of *C. mas* L. extract, regardless of its used type.

**Figure 8:**
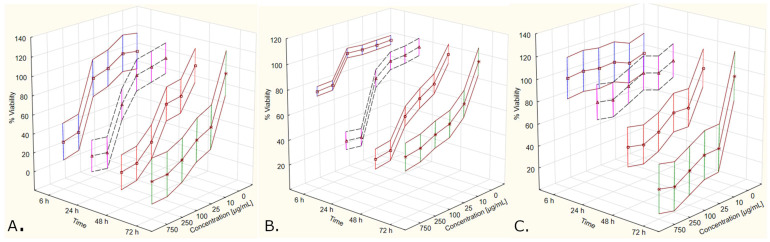
The magnitude of cytotoxicity induced with *C. mas* L. extracts on the A375 cell line, measured with use of: MTT protocol (**A**), alternative SRB protocol (**B**), standard SRB protocol (**C**). The data are shown as winsorized (95%) mean values ± standard deviation (estimated based on common variance).

**Figure 9:**
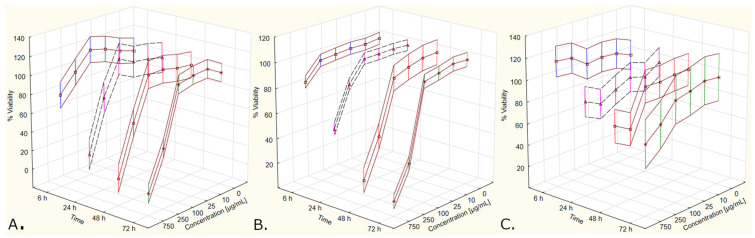
The magnitude of cytotoxicity induced with *C. mas* L. extracts on the MeWo cell line, measured with use of: MTT protocol (**A**), alternative SRB protocol (**B**), standard SRB protocol (**C**). The data are shown as winsorized (95%) mean values ± standard deviation (estimated based on common variance).

The previous sections showed that the results from the three used assay protocols led to highly similar conclusions regarding the concentration at which *C. mas* L. extracts possessed cytotoxic properties towards A375 and MeWo cells. However, as is shown in this section, the magnitude of this cytotoxicity is different for both the following cell lines: A375 (Figure 8) and MeWo (Figure 9). Results from MTT showed a greater decrease in cell viability, which could be observed even after 6 h of cell growth. The use of an alternative SRB protocol led to the same observation after 6 h of cell growth, although the inhibition of cell viability was less prominent compared to the results from the MTT assay. Interestingly, no differences in cell viability were spotted after 6 h of cell growth in the case of using the standard SRB protocol for cytotoxicity assessment. The most observable differences in cell viability measured according to this assay protocol are associated with longer cell culture times (48 h or 72 h).

**Figure 10:**
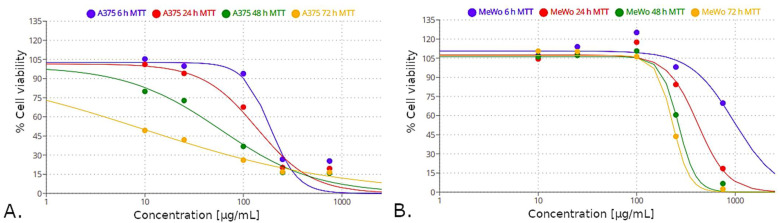
Logistic regression functions fit to the data describing: the concentration of *C. mas* L. extracts and the % of cell viability of cell lines: A375 (**A**) and MeWo (**B**) measured with the MTT method. These functions were used to calculate the IC_50_ values corresponding with each cell culture time (6 h, 24 h, 48 h, 72 h).

The differences in the size of the observed inhibitory effect of *C. mas* L. extracts in the context of different assay protocols led to different estimated values of IC_50_. For the A375 cell line, the IC_50_ values for cell culture times of the following: 6 h, 24 h, 48 h, 72 h, based on the MTT assay, were as follows: 188.67 µg/mL, 138.47 µg/mL, 58.89 µg/mL, 9.91 µg/mL, respectively (Figure 10A). MeWo cells were less susceptible to these extracts, showing IC_50_ values of the following: 970.13 µg/mL, 416.29 µg/mL, 265.47 µg/mL and 232.68 µg/mL, respectively (Figure 10B). The results from the SRB assay (regardless of the used assay protocol) may be deemed of questionable use in the context of calculating IC_50_ values since the magnitude of cytotoxic response to *C. mas* L. extracts measured with this method was markedly lower, compared to the response measured with the MTT assay (Figure 8 and Figure 9). All of the logistic regression models, along with their mathematical equations and calculated IC_50_ values (for the following three assay protocols: MTT, standard SRB and alternative SRB), are given in Appendix B (Table A5).

## 3. Discussion

### 3.1. Should the Results Be Trusted? A Brief Post-Hoc Analysis of Merits and Drawbacks of the Design of This Study and Potential Factors to Consider in Future Experiments

The hypotheses tested in this study (presented in the ‘Statistical methods’ section) were assessed with the use of multiple-way repeated measures ANOVA, which is known for its higher statistical power compared to ANOVA, allowing the analysis of smaller statistical samples while maintaining a comparatively low type I error rate. Lack of sphericity, however, inflates the type I error rate [69], increasing the odds of false-positive results. As a lack of sphericity was observed in this study, Greenhouse–Geisser and Huynh–Feldt corrections were used to decrease the type I error rate by adjusting the degrees of freedom. The factors which further increase the reliability of the results of this study are the following: the use of two different cell lines (A375 and MeWo), the count of assay methods (2 of which the MTT is deemed as ‘the gold standard’ in measuring cytotoxicity [70]), an additional alternative protocol for performing one of the assays (SRB), the count of series (4) and replications within each series (8). Interestingly, out of the two methods used in this study, the SRB method may be more suitable for experiments using compounds of oxidoreductive potential, as shown by van Tonder et al. [70].

The main problem faced in the process of data analysis was determining the presumable source of variability of the obtained results. The use of classic post-hoc tests (such as Tukey’s HSD) would provide redundant comparisons, which were not aimed to be tested *a priori* in the process of study design. Contrast analysis, used in this study, facilitated the process of hypothesis testing since it used a predefined subset of all the possible comparisons [71], allowing the analysis of a generalized growth rate trend instead of comparing the results associated with each combination of the following factors analyzed in this study: type and concentration of used Cornelian cherry extracts. This approach, however, remains not ideal in the case of this study, as the cell growth randomly varied due to conditions associated with the still-unknown action of the compounds found in the used extracts, which could not be presumed in the process of study design. This problem may be portrayed by the (control) series in which no Cornelian cherry extract was present. Due to methodological reasons, each Cornelian cherry type was ascribed to its own control series. Although these curves should hypothetically be nearly identical, slight differences could be seen at various time points. This fact might have affected the *p*-values of the F test in the case of Time*Concentration*Type interaction, showing false-positive significance. Owing to the fact that this study was aimed to provide preliminary information on the cytotoxicity of Cornelian cherry extracts towards melanoma cell lines, the authors recommend a decrease of the α-value used for statistical inference to 0.001 (instead of 0.05) so as not to over-interpret the results, especially in the section describing the contrast analysis.

Another limitation of this preliminary study may stem from the use of Cornelian cherry extracts rather than the compounds directly isolated from them. Hence, the observed cytotoxic effect, although backed up by the results of this study, remains unidentified in terms of its potential mechanism. This drawback of the study could be addressed in future experiments by assessing the concentration/activity of selected compounds found in the Cornelian Cherry extracts and using this information as a covariate factor in repeated measures analysis of covariance (ANCOVA) or using more complex statistical methods such as multivariate analysis.

It is important to note that the chemical composition of used *Cornus mas* L. extracts in the context of iridoid and phenolic content is comparable with the information provided by Dzydzan et al., where *Cornus mas* L. ‘Yantarnyi’ and ‘Podolski’ were used [50]. In the mentioned study [50], similarly to the study presented in this manuscript, anthocyanins were not detected in the yellow *Cornus mas* L. extract. Potential confusion when comparing the composition of fruits or leaves of plant species with other studies may stem from the diversity of methods used to quantify the content and the units in which some of these values are displayed [72,73,74] (for example, as gallic acid or loganic acid equivalents [24,38]). Moreover, genetic variation across *Cornus mas* L. is one of the key factors affecting the variability in the phytochemical composition of its fruits [75]. Therefore, utilizing the fruits of well-described origin is a key factor in the design of mechanistic studies associated with the action of plant nutraceuticals. In this study, authenticated voucher specimens of *Cornus mas* L. were used. Therefore, the results of this study could be referred to in future studies. More information on the differences in phytochemical content of various *Cornus mas* L. cultivars (including ‘Yantarnyi’, ‘Flava’ and ‘Podolski’, which were used in this study) could be found in a study by Kucharska et al. [24], utilizing voucher specimens. Proper storage of the fruits and extracts prevented the loss of valuable phytochemical content such as phenolics, the degradation of which has been shown to be correlated with storage temperature [76].

### 3.2. Insights into the In Vitro Antiproliferative and Cytotoxic Properties of the Cornus L. Species Based on Other Studies

As mentioned before (in the ‘Introduction’ section), the extracts obtained from the leaves and fruits of plants of the *Cornaceae* family induce both antiproliferative and cytotoxic effects on various cancer cell lines. Both of these effects contribute to the antitumor action of *Cornaceae* extracts. According to Forman et al. [77] (a study on the MCF-7 cell line), the following three *Cornus* species: *C. alba* L., *C. officinalis* L. and *C. mas* L. (used in this study) were most effective in terms of the antiproliferative action. Both the following: polyphenol and tannin content correlated with this effect. Further evidence of the antiproliferative capacity of tannins could be found in a different study in which the dimeric elagitannins of *C. alba* L. were the factors that selectively impaired proliferation of the LNCaP cell line, inducing apoptosis and S-phase arrest [78]. Yousefi et al. [58] observed the antiproliferative effect of the hydro-alcoholic extract of *C. mas* L. on the following four cancer cell lines: A549, MCF-7, SKOV3 and PC3. Regardless of the used cell line, antiproliferative effects were spotted in a broad spectrum of concentrations from 5 to 1000 µg/mL. Hosseini et al. [59] observed cytotoxic and proapoptotic effects of *C. mas* L. extract on AGS and L929 cell lines with the use of the MTT test and FITC-Annexin V binding, observed with the use of flow cytometry. Based on the figures featured in the mentioned study, the lowest concentrations of *C. mas* L. extract in which cytotoxicity could be observed were the following: 5 mg/mL (after 48 h of cell growth) or 2 mg/mL (after 72 h), regardless of the used cell line. Two other studies [36,38] showed cytotoxic activity of *C. mas* L. extract on the following various cancer cell lines: HeLa, LS174, Caco-2, HT-29, MCF-7, HepG2. In a study by Efenberger-Szmechtyk et al. [56], the cytotoxicity of *C. mas* L. leaf extracts was associated with various morphologic alterations within Caco-2 cells (chromatin condensation, cytoplasmic vacuolization, nucleus fragmentation/lysis *inter alia*). Interestingly, *C. mas* L. extract had a dichotomous effect on cell DNA, damaging it (in a dose-dependent manner) in concentrations that were associated with cytotoxic effects, or inducing DNA repair in the cells in response to hydrogen peroxide—in concentrations of the extract that did not induce cytotoxicity. Based on this study, it could be hypothesized that the compounds found in the extract exert antagonistic properties depending on their concentration. It seems likely that this effect may be associated with the antioxidative potential of these compounds since many known natural antioxidants, such as the following: phenols [79,80], anthocyanins [81], flavonoids [81,82,83,84] and carotenoids [81,85,86], may also act similar to prooxidants, depending on various conditions, such as the following: pH and their chelating behavior or solubility characteristics. This fact illustrates a potential occurrence of bias associated with drawing conclusions based solely on correlations between the antiproliferative/cytotoxic properties of plant-derived extracts and their estimated contents. Further confusion could arise upon analysis of the scientific literature discussing the topic of antiproliferative/cytotoxic effects of *C. mas* L. extracts, as both terms are often used interchangeably. Hence, many studies refer to the ‘antiproliferative effect’ while, in fact, measuring cytotoxicity with the use of assays such as MTT or SRB.

### 3.3. The Effect of Cornus mas L. Extracts on Cell Viability Observed in This Study

In most of the above-mentioned studies, only one type of *C. mas* L. was featured. The literature focuses mainly on extracts obtained from leaves or flowers, while the amount of scientific evidence regarding fruit-derived extracts remains scarce. In most studies, cell cytotoxicity was measured after 48 h or 72 h of cell growth. Moreover, none of the listed references discussed the cytotoxic effect of *C. mas* L. extracts on melanoma cell lines. In this study, the viability of two melanoma cell lines (A375, MeWo) over time under the effect of *C. mas* L. (yellow or red) fruit extracts was analyzed after 6 h, 24 h, 48 h and 72 h of growth. Analysis of these four time points as a series of data rather than independent measurements provides more insights on the studied effect.

First and foremost, it could be observed that the absolute differences in cell viability in the studied time series depended on the used assay method/protocol. The differences in the variability of the observed absorbance values measured with the MTT assay and the SRB assay stem from the fact that both assays measure different effects associated with cell viability. While the MTT method is an assessment of cell metabolism, the SRB method determines the amount of protein content. The SRB method, which was performed according to the alternative protocol, yielded lower absorbance values compared to the SRB method, to which the standard protocol was applied. This may be due to the fact that the alternative protocol included the removal of the culture medium before fixation with TCA. Thus, the proteins that were liberated from the cells during their growth or apoptosis were removed from the analyzed samples before staining with SRB. Interestingly, after removing these proteins, the SRB assay showed about 5-fold lower absorbance values compared to the MTT assay in the case of A375 cells, while the results of the same (alternative) SRB assay were over 4-fold higher compared to the MTT assay in the case of MeWo cells. Therefore, the content of proteins liberated from the cells into the culture medium during their growth/death is far greater in the case of A375 cells compared to MeWo cells. It could be hypothesized that this occurrence stems from the faster metabolism of A375 cells, as observed with the use of the MTT assay.

As mentioned before, due to the rather preliminary character of this study, an α-value of 0.001 may be more beneficial in the process of statistical inference, given that general cell viability time series (not the differences between each time point *per se*) were to be discussed in this study. If the results would be analyzed with regard to that α-value, it could be said that both SRB assay approaches revealed no significant interaction between type and concentration of *C. mas* L. extract. Results of the MTT assay would lead to the same conclusion in the case of MeWo cells but not the A375 cells. This fact may stem from different viability time series over time in the case of series in which the concentration of *C. mas* L. extracts was 10 µg/mL. In the presence of 10 µg/mL of the yellow *C. mas* L. extract, cells reached a plateau between 48 h and 72 h of growth, while they kept growing in the presence of the same concentration of red *C. mas* L. extract. As this observation is discrepant in regard to SRB assays, the hypothesis of a significant interaction between time and the type and concentration of these extracts should be updated in future research before being assumed as true. Moreover, the contrast analysis does not warrant the assumption of the said hypothesis as the studied growth time series are similar regardless of the type of used extract type. To sum it up, at this point, it is advised to view the time and concentration of *C. mas* L. extract as the factors, which affect the viability of melanoma cells. Since the type of *C. mas* L. extract did not affect the cytotoxic effect, it could be hypothesized that anthocyanin content is not associated with this effect. This hypothesis stems from the fact that one of the used extracts did not contain these compounds. This hypothesis should be tested in future studies (with the use of numerous *Cornaceae*-derived extracts of different anthocyanin content) before it may be claimed as (potentially) true in the context of cytotoxicity/impairment of proliferation induced in melanoma cells since anthocyanins (and some anthocyanin-rich extracts) were shown to induce cytotoxicity or affect the proliferation of various cancer cells [87,88,89,90,91,92,93,94].

Interesting observations could be made regarding the two cells in terms of the minimal concentrations at which the cytotoxic effect occurred. Regardless of the used assay method, it could be seen that both cell lines are of different susceptibility to the cytotoxic effect of the used extracts. Every tested concentration (range: 10 µg/mL–750 µg/mL) of the extract was cytotoxic toward A375 cells. The same conclusion could be drawn based on the three assay methods/protocols. However, the analysis of the viability of MeWo cells is more complex. Based on the results obtained with the use of the standard SRB protocol, it could be observed that *C. mas* L. extracts of concentrations within the 250 µg/mL–750 µg/mL range had a cytotoxic effect on MeWo cells. The alternative SRB and MTT assay protocols would lead to the same conclusion. However, if a standard α-value of 0.05 was used for statistical inference, it could be hypothesized that 100 µg/mL may also, although mildly, have had a transient cytotoxic effect on MeWo cells.

In the previous section, the cytotoxic and antiproliferative actions of *Cornus* L. extracts were presented in reference to other studies. In this study, in one of the MeWo time series (750 µg/mL of *C. mas* L. extract) obtained with the use of the MTT assay, cell metabolism decreased with time. The respective time series (750 µg/mL of *C. mas* L. extract) obtained with the use of the SRB assay (alternative protocol) showed the same occurrence (decrease in absorbance over time). Interestingly, some of the time series (such as the one associated with 250 µg/mL of *C. mas* L. extract, obtained with the use of an alternative SRB assay protocol) showed a markedly decreased rate of cell growth (a mild increase in absorbance) compared to the control time series. Thus, both cytotoxic and antiproliferative effects could be hypothesized with regard to the cell viability time series featured in this study.

An interesting observation was made after transforming the results from raw absorbance values into the percentage of cell viability so as to calculate IC_50_ values. The MTT assay revealed a higher relative cytotoxic response of both cell lines to *C. mas* L. extracts compared to the results obtained with the SRB assay, regardless of the used assay protocol. Moreover, the SRB assay showed higher values of the aforementioned cell response when the alternative assay protocol was applied. Regardless of the used cell line, no cytotoxic response to *Cornus mas* L. was observed with the SRB assay after 6 h of cell culture. These facts affected the IC_50_ values estimated with the use of logistic regression models, rendering some of these values (namely, those associated with the ‘standard’ SRB assay, after 6 h of cell culture, regardless of the cell line) non-computable. These observations may presumably stem from the different nature of both these assays. Since metabolic changes are spotted earlier than the factual cell lysis, the MTT assay (which assesses the cell metabolic activity) provided markedly lower IC_50_ values compared to SRB (used to determine cellular protein content). Interestingly, IC_50_ values associated with the MTT assay could account for the fact that MeWo cells are less susceptible to *C. mas* L. extracts compared to the A375 cells, as shown based on the growth time series analyzed in this study. The IC_50_ values estimated in this study should rather be perceived as preliminary, providing the grounds for future research on this matter.

Although no other study found in the literature covers the exact problem discussed in this study, there is evidence that MeWo and A375 cells differ from each other (or from primary melanocytes in general) in terms of cytotoxicity or proliferation. Qiao et al. [95] observed that A375 cells were susceptible to the pro-oxidative action of thiostrepton. Oxidative stress in these cells evoked upregulation of heat shock protein expression and apoptotic and proteogenic effects. This effect was antagonized by antioxidative treatment. Interestingly, primary melanocytes were not affected by thiostrepton. The higher susceptibility of melanoma cells to oxidative stress may presumably stem from alterations in antioxidative mechanisms within these cells in comparison to primary melanocytes. The expression of one of the S100 proteins, S100A10 (hypothesized to be associated with cell proliferation [96]), was downregulated in three melanoma cell lines (G-361, A375 and MeWo) compared to normal melanocytes (HEMn cell line). Of the three melanoma cell lines, MeWo showed higher S100A10 expression [96]. Okazawa et al. [97] observed that out of three melanoma cell lines (A375, MeWo, HM3KO), only A375 was prone to growth inhibition by endothelin-1. The fact that melanoma cells may be selectively affected by specific antiproliferative/cytotoxic agents is promising in terms of the future development of cancer treatment.

Despite its limitations, this study shows that fruit extracts of yellow or red *C. mas* L. have a cytotoxic effect on the following two melanoma cell lines: A375 and MeWo. There is no sufficient evidence to claim that the type of the used extract induced a different cytotoxic effect in the tested cell lines. Interestingly, the A375 cell line was more prone to cytotoxicity compared to MeWo cells. These results may also imply that other melanoma cells may also differ in susceptibility to *C. mas* L. extracts and, perhaps, to extracts derived from other species of the *Cornaceae* family. Future tests may need to feature a greater number of tested melanoma cell lines to examine the patomechanism of the cytotoxicity of *C. mas* L. extracts. Examining the potentially variable antioxidative capacity of melanoma cells may be of significance in the context of the development of new hypotheses regarding the susceptibility of melanoma cells to cytotoxic effects, potentially providing novel solutions in the utilization of plant-based extracts (or their compounds) in targeted, anti-cancer treatment.

## 4. Materials and Methods

### 4.1. The Procurement of the Material, Its Identification and Quantitative and Qualitative Characterization

All reagents and organic solvents were of analytical grade. Authentic standards of loganic acid, cyanidin 3-*O*-glucoside, p-coumaric acid, gallic acid, quercetin 3-*O*-glucoside, kaempferol 3-*O*-glucoside were purchased from Extrasynthese (Genay, France). Trans-caftaric acid was purchased from Cayman Chemical Company (Michigan, EUA, Ann Arbor, MI, USA). Trans-coutaric acid was purchased from Merck (Darmstadt, Germany). Methanol, acetonitrile and formic acid were obtained from POCh (Gliwice, Poland).

#### 4.1.1. Preparation and Purification of Extracts 

Yellow (‘Yantarnyi’ and ‘Flava’) and red (‘Podolski’) cornelian cherry fruits (*Cornus mas* L.) were harvested from the Arboretum in Bolestraszyce, near Przemyśl, Poland. The plant materials were authenticated by Elżbieta Żygała, M.Sc. (Arboretum and Institute of Physiography in Bolestraszyce, Przemyśl, Poland), and the adequate voucher specimens (‘Yantarnyi’—BDPA 14131; ‘Flava’—BDPA 8795; ‘Podolski’—BDPA 10462) have been deposited at the Herbariums of Arboretum in Bolestraszyce, Poland. After harvesting fruits were immediately frozen at −20 °C. Frozen ripe fruits of cornelian cherry were shredded and heated for 5 min at 95 °C using a Thermomix (Vorwerk, Wuppertal, Germany). The pulp was subsequently cooled down to 50 °C and depectinized at 50 °C for 2 h by adding 0.5 mL of Pectinex BE XXL (Novozymes A/S, Denmark) per 1 kg. After depectinization, the pulp was pressed in a laboratory hydraulic press (SRSE, Warsaw, Poland). The pressed juice was filtered and run through an Amberlite XAD-16 resin column (Rohm and Haas, Chauny Cedex, France) for purification. Impurities (sugars and organic acids) were washed off with distilled water. During the washing of the column with water, the process was monitored on an ongoing basis (with use of HPLC) and no losses of water-soluble bioactive compounds were observed. Two purified extracts (one from yellow *C. mas* L. and one from red *C. mas* L.) were eluted with 80% ethanol. The extracts were concentrated under vacuum at 40 °C. The solvent was evaporated using a Rotavapor (Unipan, Warsaw, Poland) and then the extracts were freeze-dried (Alpha 1–4 LSC, Christ, Osterode am Harz, Germany).

#### 4.1.2. Qualitative Identification by Means of LC-MS

The method was previously described by Przybylska et al. [68]. Identification of compounds was carried out via the Acquity ultra-performance liquid chromatography (UPLC) system, coupled with a quadrupole-time of flight (Q-TOF) MS instrument (UPLC/Synapt Q-TOF MS, Waters Corp., Milford, MA, USA), with an electrospray ionization (ESI) source. Separation was achieved on an Acquity UPLC BEH C18 column (100 × 2.1 mm i.d., 1.7 µm; Waters Corp., Milford, MA, USA). The mobile phase was composed of a mixture of 2.0% aq. Formic acid *v*/*v* (A) and acetonitrile (B). The following gradient program was used: initial conditions, 1% B in A; 12 min, 25% B in A; 12.5 min, 100% B; 13.5 min, 1% B in A. The flow rate was 0.45 mL/min, and the injection volume was 5 µL. The column was operated at 30 °C. UV-Vis absorption spectra were recorded online during UPLC analysis, and the spectral measurements were made in the wavelength range of 200–600 nm, in steps of 2 nm. The major operating parameters for the Q-TOF MS were set as follows: capillary voltage 2.0 kV, cone voltage 40 V, cone gas flow of 11 L/h, collision energy 28–30 eV, source temperature 100 °C, desolvation temperature 250 °C, collision gas, argon; desolvation gas (nitrogen) flow rate, 600 L/h; data acquisition range, *m*/*z* 100–2500 Da. The compounds were monitored at 245, 280, 320, 360, 520 nm and explored in the negative and positive (in case of anthocyanins) modes before and after fragmentation. The data were collected with Mass-Lynx V 4.1 software (Waters Corp., Milford, MA, USA).

#### 4.1.3. Quantitative Determination of Anthocyanins, Flavonols, Phenolic Acids and Iridoids by HPLC-PDA

The HPLC analysis was carried out according to Spychaj et al. [98] using a Dionex (Germering, Germany) system equipped with diode array detector Ultimate 3000, quaternary pump LPG-3400A, autosampler EWPS-3000SI, thermostated column compartment TCC-3000SD and controlled by Chromeleon v.7.2 software. Separation was achieved using a Cadenza Imtakt column CD-C18 (75 × 4.6 mm, 5 μm). The mobile phase was composed of solvent A (4.5% aq. formic acid, *v*/*v*) and solvent B (100% acetonitrile). The gradient profile was as follows: 0–1 min 5% B in A, 1–20 min 25% B in A, 20–26 min 100% B, 26–30 min 5% B in A. The flow rate of the mobile phase was 1 mL/min, and the injection volume was 20 μL. The column was operated at 30 °C. Anthocyanins were detected at 520 nm, flavonols at 360 nm, phenolic acids at 320 nm and iridoids at 245 nm. Calibration curves at concentrations in range of 0.02–0.3 mg/mL (R2 ≥ 0.9998) were determined experimentally for cyanidin 3-*O*-glucoside, quercetin 3-*O*-glucoside, kaempferol 3-*O*-glucoside, caffeic acid and p-coumaric acid. The results were provided as mean ± standard deviation from three replications and expressed as milligrams per 100 g of the dry extract.

#### 4.1.4. Quantitative Determination of Hydrolyzable Tannins by HPLC-PDA

The HPLC analysis was performed according to Przybylska et al. [68] using a Dionex (Germering, Germany) system equipped with diode array detector Ultimate 3000, quaternary pump LPG-3400A, autosampler EWPS-3000SI, thermostated column compartment TCC-3000SD and controlled by Chromeleon v.7.2 software. Separation was achieved on a Hypersil GOLD C18-column (250 × 4.6 mm, 5 μm; Thermo Fisher Scientific Inc., Leicestershire, UK). The following mixtures were used as eluents: A, water-FA (98.5:1.5, *v*/*v*) and DB, acetonitrile-FA (98.5:1.5, *v*/*v*). The following gradient profile was applied: initial conditions 100% A, 30 min; 30% B, 33 min; 70% B, 45 min; 70% B in A, 48 min; 100% B, 55–60 min; 100% A. The flow rate of the mobile phase was 1.2 mL/min, and the injection volume was 20 μL. The column was operated at 22 °C. Hydrolyzable tannins were detected at 280 nm. Calibration curve at concentrations in range of 0.02–0.3 mg/mL (R2 ≥ 0.9996) was determined experimentally for gallic acid. Results are provided as the total of individual isomers of three replications and expressed as milligrams per 100 g of the dry extract.

### 4.2. Cell Viability Assays

#### 4.2.1. Cell Culture

Human melanoma cell lines—MeWo (ATCC^®^ HTB-65™) and A375 (ATCC^®^ CRL-1619™) were purchased from American Type Culture Collection (ATCC; Manassas, VA, USA). MeWo cells were cultured in culture flasks (T-75, Falcon^®^, Corning Life Sciences, Tewksbury, MA, USA) in Minimum Essential Medium (MEM; without phenol red; Gibco, Thermo Fisher Scientific, Waltham, MA, USA) supplemented with 2 mM of GlutaMAX™ (Gibco, Thermo Fisher Scientific, Waltham, MA, USA), 1 mM sodium pyruvate solution (Sigma-Aldrich, Saint Louis, MO, USA), MEM Non-Essential Amino Acid Solution (Sigma-Aldrich, Saint Louis, MO, USA). A375 cells were cultured in Dulbecco’s Modified Eagle Medium (DMEM; without phenol red, Gibco, Thermo Fisher Scientific, Waltham, MA, USA), respectively. Cell culture media were supplemented with 10% heat-inactivated fetal bovine serum (FBS; Gibco, Thermo Fisher Scientific, Waltham, MA, USA) and 1% stabilized antibiotic antimycotic solution containing 10,000 units of penicillin/mL, 10 mg/mL of streptomycin and 25 µg/mL of amphotericin B (Sigma-Aldrich, Saint Louis, MO, USA). The medium was renewed every 3 days. The cells were cultured under standard culture conditions at 37 °C in humidified air containing 5% CO_2_ in a CELCULTURE^®^ CCL-170B-8 incubator (Esco Micro Pte Ltd., Singapore). For experiments, the cells were harvested with TrypLE™ Express (Gibco, Thermo Fisher Scientific, Waltham, MA, USA), stained with 0.4% trypan blue solution and counted with use of Countess™ Automated Cell Counter (Invitrogen, Thermo Fisher Scientific, Waltham, MA, USA).

In total, 200 µL of medium with suspended cells were placed in each well of a 96-well microtiter plate (Eppendorf AG, Hamburg, Germany). Each well initially contained 1.0 × 10^4^ or 5.0 × 10^3^ cells. After seeding, cells were maintained for 24 h in a CO_2_ incubator for cell attachment and homeostasis. Next, the cell culture medium was withdrawn from the wells and replaced with 200 µL of fresh cell culture medium with addition of red or yellow Cornelian cherry extract. Stock aqueous solutions (10 mg/mL) of extracts were used for further dilutions. The concentration of the extracts was 10, 100, 250 or 750 µg/mL. This experiment was performed in four series utilizing cells from different cell passages. Each series consisted of 8 replicates corresponding to different growth conditions (variable concentration and type of the Cornelian cherry extract).

#### 4.2.2. Cytotoxicity Measurements with Use of the MTT Method

The culture medium was removed from the wells and 100 μL of 0.5 mg/mL MTT (3-(4,5-dimethylthiazol-2-yl)-2,5-diphenyltetrazolium bromide, Sigma-Aldrich, Saint Louis, MO, USA) solution in PBS buffer was added. After 2 h incubation at 37 °C, acidified isopropanol (100 μL, 0.04 M HCl in 99.9% isopropanol) was added to dissolve formazan crystals. Absorbance was measured at 570 nm using the multiplate reader (GloMax^®^, Promega GmbH, Walldorf, Germany).

After 6, 24, 48 and 72 h of treatment, post-culture medium was removed, cells were rinsed with sterile PBS solution. Then, 100 μL of 0.5 mg/mL 3-(4,5-dimethylthiazol-2-yl)-2,5-diphenyl tetrazolium bromide in complete growth medium (MTT reagent; Sigma-Aldrich, Saint Louis, MO, USA) was added. Microtiter plates were incubated for 3 h in the CO_2_ incubator under the aforementioned conditions. Subsequently, the MTT reagent was decanted, and the formed formazan crystals were dissolved in dimethyl sulfoxide (DMSO; BioShop, Burlington, ON, Canada). The absorbance was measured using an Infinite^®^ M200 plate spectrophotometer (Tecan Group Ltd., Männedorf, Switzerland) at λ = 540 nm.

#### 4.2.3. Cytotoxicity Measurements with Use of the SRB Method

After the 6, 24, 48 and 72 h incubation periods, post-culture medium was removed and cells were washed with sterile phosphate-buffered saline (PBS) solution (‘alternative’ protocol) or left to stand (‘standard’ protocol, according to the literature [99]). Subsequently, TCA (trichloroacetic acid) was used for fixation. The final concentration of TCA was 10%. After 1 h incubation at +4 °C, the cells were washed at least 5 times with distilled water and dried. Then, a freshly prepared solution of 0.04% SRB (Sigma-Aldrich, USA) in 1% acetic acid (Avantor Performance Materials Poland, Gliwice, Poland) was added to each well and the plates were left at room temperature, in the dark, for 30 min. Subsequently, the dye was removed from each well and the microtiter plates were washed in 1% acetic acid so as to remove the excess dye. The SRB, which remained after the washing was solubilized in 10 mM Tris base solution (pH 10.5). The absorbance (proportional to the protein content within the cells) was measured using an Infinite^®^ M200 plate spectrophotometer (Tecan Group Ltd., Männedorf, Switzerland) at λ = 520 nm.

### 4.3. Statistical Methods

Statistical analysis was performed with use of STATISTICA 13.3. package (StatSoft, Poland, Kraków, Poland) on license by Wroclaw Medical University. Multiple-way repeated measures analysis of variance (Multiple-way RM-ANOVA) with σ-restricted parametrization was used to check for significance of ‘Time’ and the following two other variables: the type of used Cornelian cherry extract (referred to as ‘Type’) and the concentration of the used extract (‘Concentration’). Between-variable interactions (Time*Type, Time*Concentration, Time*Type*Concentration) were also tested. Mauchly’s test was used to test for sphericity, although due to the lack of sphericity (Appendix A, Table A1), degrees of freedom were adjusted with use of Greenhouse–Geisser and Huynh–Feldt corrections, separately.

As the analysis was aimed to evaluate cell growth trend over time (not the quantity of the cells between each time point), contrast analysis was employed to compare the growth trend between the different sets of measurements (associated with different Cornelian cherry extract types and concentrations). The used set of hypotheses for contrast analysis was optimal for exploratory data analysis. The main hypotheses tested in this study were as follows:

I. There is at least one concentration in which Cornelian cherry extract(s) have a cytotoxic effect over the analyzed melanoma cell line(s);
II. The overall cell growth trend will be unaffected by the type of Cornelian cherry extract(s), under their presence in the cell culture medium;

These hypotheses were evaluated with use of two conjoined sets of *a priori*, auxiliary hypotheses (being a part of the contrast analysis) testing for equality of mean values as follows:

I. Comparisons between series of measurements associated with different concentrations of Cornelian cherry extracts as follows (contrasts):
  - (C1) Control series vs. series with concentration equal to 10 µg/mL;
  - (C2) Control series vs. series with concentration equal to 25 µg/mL;
  - (C3) Control series vs. series with concentration equal to 100 µg/mL;
  - (C4) Control series vs. series with concentration equal to 250 µg/mL;
  - (C5) Control series vs. series with concentration equal to 750 µg/mL.;
II. Comparisons between time points (hypotheses for each contrast according to Helmert coding matrix as follows [100,101]):
  - (M1) 6th hour of growth vs. other time points (24th hour, 48th hour, 72nd hour);
  - (M2) 24th hour of growth vs. the two next time points (48th hour, 72nd hour);
  - (M3) 48th hour of growth vs. the last time point (72nd hour).

As an example, a “C3-M2” set of hypotheses was used to check whether there was a significant difference between control series and series in which the concentration of Cornelian cherry extract was 100 µg/mL. The analyzed difference between time points in that comparison was 24th vs. (48th + 72nd) hours of cell growth. The described procedures facilitated the evaluation of the curve of cell growth, accounting for the fact that the increase in cell count over time has its limit. Contrast analysis was performed separately for two different types of Cornelian cherry extract. Additionally in the last ‘Results’ subsection, as the means for preventing drawing false conclusions from this study, α = 0.001 is discussed as the cut-off value for statistical inference apart from the commonly used α = 0.05. Both values are referred to in the text—to provide additional insights into the data.

IC_50_ was calculated based on three-parameter logistic regression [102]. For this purpose, the absorbance values were transformed into % of cell viability as series associated with each time of cell culture (6 h, 24 h, 48 h, 72 h).

## 5. Conclusions

The following conclusions could be drawn from this study:

- Extracts of yellow and red *Cornus mas* L. exert cytotoxic properties towards the following melanoma cell lines: A375 and MeWo;
- The A375 cell line was more susceptible to the cytotoxic effect of the *Cornus mas* L. extracts compared to the MeWo cell line.

The following hypotheses need more evidence before they may be claimed as valid:

- Cytotoxic properties of *Cornus mas* L. extracts do not differ in the context of the type of extract (whether it was collected from red or yellow *Cornus mas* L. species);
- Anthocyanin content is not associated with the cytotoxic properties of *Cornus mas* L. extract towards melanoma cell lines (since the two extracts induced the same cytotoxic effect and one of them did not contain anthocyanins).

## Figures and Tables

**Table 1:** Results of the analysis of interactions performed on various datasets of this study.

Dataset	Effect	Unadj. df	F	GG ε	GG adj. df_effect_	GG *p*	HF ε	HF adj. df_effect_	HF *p*	Sign.
A375, SRB, alternative	Time	3.00	56.90	0.5430	1.63	<0.00001	0.5612	1.68	<0.00001	**
	Time*Type	3.00	18.92	0.5430	1.63	<0.00001	0.5612	1.68	<0.00001	**
	Time*Concentration	15.00	79.25	0.5430	8.14	<0.00001	0.5612	8.42	<0.00001	**
	Time*Type*Concentration	15.00	1.85	0.5430	8.14	0.0642	0.5612	8.42	0.0617	
A375, SRB, standard	Time	3.00	282.99	0.3945	1.18	<0.00001	0.4067	1.22	<0.00001	**
	Time*Type	3.00	0.33	0.3945	1.18	0.6054	0.4067	1.22	0.6122	
	Time*Concentration	15.00	92.25	0.3945	5.92	<0.00001	0.4067	6.10	<0.00001	**
	Time*Type*Concentration	15.00	0.73	0.3945	5.92	0.6241	0.4067	6.10	0.6282	
MeWo, SRB, alternative	Time	3.00	4612.49	0.4770	1.43	<0.00001	0.4925	1.48	<0.00001	**
	Time*Type	3.00	1.39	0.4770	1.43	0.2476	0.4925	1.48	0.2481	
	Time*Concentration	15.00	448.08	0.4770	7.16	<0.00001	0.4925	7.39	<0.00001	**
	Time*Type*Concentration	15.00	1.62	0.4770	7.16	0.1249	0.4925	7.39	0.1222	
MeWo, SRB, standard	Time	3.00	1614.87	0.4743	1.42	<0.00001	0.4896	1.47	<0.00001	**
	Time*Type	3.00	6.45	0.4743	1.42	0.0051	0.4896	1.47	0.0047	*
	Time*Concentration	15.00	26.92	0.4743	7.11	<0.00001	0.4896	7.34	<0.00001	**
	Time*Type*Concentration	15.00	2.36	0.4743	7.11	0.0213	0.4896	7.34	0.0199	*
A375, MTT	Time	3.00	539.05	0.5961	1.79	<0.00001	0.6237	1.87	<0.00001	**
	Time*Type	3.00	3.40	0.5961	1.79	0.0393	0.6237	1.87	0.0371	*
	Time*Concentration	15.00	256.34	0.5961	8.94	<0.00001	0.6237	9.36	<0.00001	**
	Time*Type*Concentration	15.00	5.74	0.5961	8.94	<0.00001	0.6237	9.36	<0.00001	**
MeWo, MTT	Time	3.00	405.96	0.5409	1.62	<0.00001	0.5590	1.68	<0.00001	**
	Time*Type	3.00	3.03	0.5409	1.62	0.0598	0.5590	1.68	0.0581	
	Time*Concentration	15.00	85.16	0.5409	8.11	<0.00001	0.5590	8.39	<0.00001	**
	Time*Type*Concentration	15.00	2.71	0.5409	8.11	0.0059	0.5590	8.39	0.0053	*

Abbreviations: ‘Unadj. df’, unadjusted degrees of freedom; ‘GG’, Greenhouse–Geisser correction; ‘HF’, Huynh–Feldt correction; ’adj. df_effect_’, adjusted (GG or HF) degrees of freedom for the effect/interaction; ‘sign.’, significance (marked as: ‘*’ if *p* ∈ [0.001; 0.05) or ‘**’ if *p* < 0.001).

**Table 2:** Results of the contrast analysis, performed in various datasets of this study.

		Type: Yellow			Type: Red		
Dataset	Hypothesis	M1	M2	M3	M1	M2	M3
A375, SRB, alternative	C1	<0.00001	<0.00001	<0.00001	<0.00001	<0.00001	<0.00001
	C2	<0.00001	<0.00001	<0.00001	<0.00001	<0.00001	<0.00001
	C3	<0.00001	<0.00001	<0.00001	<0.00001	<0.00001	<0.00001
	C4	<0.00001	<0.00001	0.005379	<0.00001	<0.00001	0.13513
	C5	<0.00001	<0.00001	0.000054	<0.00001	<0.00001	0.49060
Dataset	Hypothesis	M1	M2	M3	M1	M2	M3
A375, SRB, standard	C1	<0.00001	<0.00001	<0.00001	<0.00001	<0.00001	<0.00001
	C2	<0.00001	<0.00001	<0.00001	<0.00001	<0.00001	<0.00001
	C3	<0.00001	<0.00001	<0.00001	<0.00001	<0.00001	<0.00001
	C4	<0.00001	<0.00001	<0.00001	<0.00001	<0.00001	<0.00001
	C5	<0.00001	<0.00001	<0.00001	<0.00001	<0.00001	<0.00001
Dataset	Hypothesis	M1	M2	M3	M1	M2	M3
MeWo, SRB, alternative	C1	0.37012	0.42344	0.13206	0.78527	0.73135	0.71821
	C2	0.67862	0.89692	0.73977	0.36564	0.17724	0.95834
	C3	0.56375	0.03943	0.02891	0.16974	0.04503	0.00175
	C4	<0.00001	<0.00001	<0.00001	<0.00001	<0.00001	<0.00001
	C5	<0.00001	<0.00001	<0.00001	<0.00001	<0.00001	<0.00001
Dataset	Hypothesis	M1	M2	M3	M1	M2	M3
MeWo, SRB, standard	C1	0.40450	0.05480	0.09584	0.67448	0.06699	0.05762
	C2	0.27217	0.16651	0.66909	0.01365	0.96974	0.15757
	C3	0.56117	0.06853	0.95977	0.06680	0.73241	0.98845
	C4	<0.00001	0.01703	0.10514	<0.00001	0.00002	0.00002
	C5	0.00009	0.01035	<0.00001	<0.00001	<0.00001	0.00045
Dataset	Hypothesis	M1	M2	M3	M1	M2	M3
A375, MTT	C1	<0.00001	<0.00001	<0.00001	<0.00001	<0.00001	<0.00001
	C2	<0.00001	<0.00001	<0.00001	<0.00001	<0.00001	<0.00001
	C3	<0.00001	<0.00001	<0.00001	<0.00001	<0.00001	<0.00001
	C4	<0.00001	<0.00001	<0.00001	<0.00001	<0.00001	<0.00001
	C5	<0.00001	<0.00001	<0.00001	<0.00001	<0.00001	<0.00001
Dataset	Hypothesis	M1	M2	M3	M1	M2	M3
MeWo, MTT	C1	0.73106	0.10687	0.64271	0.05644	0.55723	0.17045
	C2	0.49058	0.27445	0.23698	0.10326	0.96196	0.04834
	C3	0.00661	0.27255	0.02596	0.01786	0.52584	0.44752
	C4	<0.00001	<0.00001	<0.00001	<0.00001	<0.00001	0.18981
	C5	<0.00001	<0.00001	0.02284	<0.00001	<0.00001	0.42426

Values in the brackets represent respective *p*-values for each set of conjoined hypotheses (C1–C5; M1–M3) described in the ‘Statistical methods’ section. ‘Type’ indicates the type of *Cornus mas* extract used in the experimental series. *p*-values < 0.05 were colored. The darker color marks *p* < 0.001.

## Data Availability

Not applicable.

## Supplementary Materials

The following supporting information can be downloaded at: https://www.mdpi.com/article/10.3390/molecules27134193/s1. Table S1: Identification and content (mg/100 g dry weight (dw)) of main compounds of extracts from yellow and red Cornelian cherry (*Cornus mas* L.) fruits by means of LC-MS and HPLC; Figure S1: Cell protein content curves (A375 cell line, SRB assay) in the context of different types (Time*Type interaction, A) and concentrations of Cornelian cherry extract (Time*Concentration interaction, B). The values were obtained with use of the alternative assay protocol. The values are given as estimated marginal means ± standard error; Figure S2: Cell protein content curves (A375 cell line, SRB assay) in context of different types (Time*Type interaction, A) and concentrations of Cornelian cherry extract (Time*Concentration interaction, B). The values were obtained with use of the standard assay protocol. The values are given as estimated marginal means ± standard error; Figure S3: Cell protein content curves (MeWo cell line, SRB assay) in the context of different types (Time*Type interaction, A) and concentrations of Cornelian cherry extract (Time*Concentration interaction, B). The values were obtained with use of the alternative assay protocol. The values are given as estimated marginal means ± standard error; Figure S4: Cell protein content curves (MeWo cell line, SRB assay) in the context of different types (Time*Type interaction, A) and concentrations of Cornelian cherry extract (Time*Concentration interaction, B). The values were obtained with use of the standard assay protocol. The values are given as estimated marginal means ± standard error; Figure S5: Metabolic activity curves (A375 cell line, MTT assay) in the context of different types (Time*Type interaction, A) and concentrations of Cornelian cherry extract (Time*Concentration interaction, B). The values are given as estimated marginal means ± standard error; Figure S6: Metabolic activity curves (MeWo cell line, MTT assay) in the context of different types (Time*Type interaction, A) and concentrations of Cornelian cherry extract (Time*Concentration interaction, B). The values are given as estimated marginal means ± standard error.

## Sample Availability

Samples of featured *Cornus mas* L. extracts are available from the authors.

## Appendix A

**Table A1:** Results of Mauchly’s W test for data sphericity in datasets analyzed in this study.

Dataset	Effect	W	χ2	df	*p*
A375, SRB, alternative	Time	0.1953	623.53	5	<0.00001
A375, SRB, standard	Time	0.2492	381.78	5	<0.00001
MeWo, SRB, alternative	Time	0.0030	2216.60	5	<0.00001
MeWo, SRB, standard	Time	0.2365	534.46	5	<0.00001
A375, MTT	Time	0.0368	1224.43	5	<0.00001
MeWo, MTT	Time	0.1613	676.32	5	<0.00001

**Table A2:** Descriptive statistics of the expected marginal values of absorbance (λ = 520 nm) associated with measurements under the exposition to different types of *Cornus mas* L. extract (Time*Type interaction), measured with use of various assays.

A375, SRB, Alternative						
Type	Time	Mean Value	SE	−95% CI	95% CI	N
yellow	6 h	0.1228	0.0018	0.1192	0.1264	192
yellow	24 h	0.1399	0.0024	0.1351	0.1447	192
yellow	48 h	0.1415	0.0035	0.1347	0.1484	192
yellow	72 h	0.1310	0.0035	0.1241	0.1380	192
red	6 h	0.1188	0.0018	0.1152	0.1223	192
red	24 h	0.1299	0.0024	0.1252	0.1347	192
red	48 h	0.1394	0.0035	0.1325	0.1463	192
red	72 h	0.1448	0.0035	0.1379	0.1517	192
A375, SRB, standard						
Type	Time	Mean value	SE	−95% CI	95% CI	N
yellow	6 h	0.1238	0.0048	0.1143	0.1333	192
yellow	24 h	0.1535	0.0082	0.1375	0.1695	192
yellow	48 h	0.2119	0.0118	0.1887	0.2351	192
yellow	72 h	0.3908	0.0241	0.3434	0.4382	192
red	6 h	0.1067	0.0048	0.0972	0.1162	192
red	24 h	0.1563	0.0082	0.1402	0.1723	192
red	48 h	0.2030	0.0118	0.1799	0.2262	192
red	72 h	0.3848	0.0241	0.3374	0.4323	192
MeWo, SRB, alternative						
Type	Time	Mean value	SE	−95% CI	95% CI	N
yellow	6 h	0.1744	0.0024	0.1697	0.1791	192
yellow	24 h	0.2539	0.0033	0.2475	0.2603	192
yellow	48 h	0.3515	0.0070	0.3377	0.3653	192
yellow	72 h	0.5826	0.0088	0.5653	0.5999	192
red	6 h	0.1647	0.0024	0.1600	0.1693	192
red	24 h	0.2375	0.0033	0.2311	0.2440	192
red	48 h	0.3460	0.0070	0.3322	0.3598	192
red	72 h	0.5637	0.0088	0.5464	0.5810	192
MeWo, SRB, standard						
Type	Time	Mean value	SE	−95% CI	95% CI	N
yellow	6 h	0.1994	0.0042	0.1912	0.2076	192
yellow	24 h	0.3046	0.0070	0.2909	0.3183	192
yellow	48 h	0.4410	0.0103	0.4207	0.4613	192
yellow	72 h	0.9286	0.0225	0.8844	0.9728	192
red	6 h	0.1863	0.0042	0.1781	0.1945	192
red	24 h	0.2554	0.0070	0.2417	0.2691	192
red	48 h	0.3409	0.0103	0.3205	0.3612	192
red	72 h	0.8425	0.0225	0.7983	0.8867	192
A375, MTT						
Type	Time	Mean value	SE	−95% CI	95% CI	N
yellow	6 h	0.0642	0.0023	0.0596	0.0687	192
yellow	24 h	0.1180	0.0036	0.1110	0.1250	192
yellow	48 h	0.1530	0.0058	0.1415	0.1645	192
yellow	72 h	0.2033	0.0065	0.1905	0.2161	192
red	6 h	0.0585	0.0023	0.0539	0.0630	192
red	24 h	0.1062	0.0036	0.0992	0.1132	192
red	48 h	0.1554	0.0058	0.1439	0.1668	192
red	72 h	0.2144	0.0065	0.2016	0.2272	192
MeWo, MTT						
Type	Time	Mean value	SE	−95% CI	95% CI	N
yellow	6 h	0.1181	0.0027	0.1129	0.1233	192
yellow	24 h	0.1570	0.0029	0.1513	0.1628	192
yellow	48 h	0.2062	0.0038	0.1987	0.2138	192
yellow	72 h	0.2020	0.0052	0.1919	0.2122	192
red	6 h	0.1084	0.0027	0.1032	0.1136	192
red	24 h	0.1431	0.0029	0.1373	0.1488	192
red	48 h	0.1813	0.0038	0.1738	0.1889	192
red	72 h	0.1906	0.0052	0.1804	0.2008	192

**Table A3:** Descriptive statistics of the expected marginal values of absorbance (λ = 520 nm) associated with measurements under the exposition to different concentration of *Cornus mas* L. extracts (Time*Concentration interaction), measured with use of various assays.

A375, SRB, Alternative						
Concentration [µg/mL]	Time	Mean Value	SE	−95% CI	95% CI	N
0	6 h	0.1302	0.0032	0.1240	0.1364	64
0	24 h	0.1738	0.0042	0.1655	0.1821	64
0	48 h	0.2206	0.0060	0.2087	0.2325	64
0	72 h	0.2335	0.0061	0.2215	0.2454	64
10	6 h	0.1300	0.0032	0.1238	0.1362	64
10	24 h	0.1706	0.0042	0.1623	0.1789	64
10	48 h	0.1753	0.0060	0.1634	0.1872	64
10	72 h	0.1562	0.0061	0.1442	0.1682	64
25	6 h	0.1298	0.0032	0.1236	0.1361	64
25	24 h	0.1713	0.0042	0.1630	0.1796	64
25	48 h	0.1578	0.0060	0.1459	0.1697	64
25	72 h	0.1271	0.0061	0.1151	0.1390	64
100	6 h	0.1335	0.0032	0.1272	0.1397	64
100	24 h	0.1530	0.0042	0.1447	0.1613	64
100	48 h	0.1404	0.0060	0.1286	0.1523	64
100	72 h	0.1240	0.0061	0.1120	0.1360	64
250	6 h	0.1017	0.0032	0.0955	0.1079	64
250	24 h	0.0695	0.0042	0.0612	0.0778	64
250	48 h	0.0758	0.0060	0.0639	0.0877	64
250	72 h	0.0921	0.0061	0.0802	0.1041	64
750	6 h	0.0996	0.0032	0.0934	0.1059	64
750	24 h	0.0712	0.0042	0.0629	0.0795	64
750	48 h	0.0728	0.0060	0.0609	0.0846	64
750	72 h	0.0947	0.0061	0.0827	0.1067	64
A375, SRB, standard						
Concentration [µg/mL]	Time	Mean value	SE	−95% CI	95% CI	N
0	6 h	0.1150	0.0084	0.0986	0.1315	64
0	24 h	0.1752	0.0141	0.1475	0.2030	64
0	48 h	0.3549	0.0204	0.3147	0.3950	64
0	72 h	1.0839	0.0418	1.0017	1.1661	64
10	6 h	0.1040	0.0084	0.0875	0.1204	64
10	24 h	0.1619	0.0141	0.1342	0.1897	64
10	48 h	0.2495	0.0204	0.2093	0.2896	64
10	72 h	0.4138	0.0418	0.3316	0.4960	64
25	6 h	0.1244	0.0084	0.1080	0.1409	64
25	24 h	0.1956	0.0141	0.1678	0.2233	64
25	48 h	0.2252	0.0204	0.1850	0.2653	64
25	72 h	0.3551	0.0418	0.2729	0.4373	64
100	6 h	0.1210	0.0084	0.1046	0.1375	64
100	24 h	0.1516	0.0141	0.1238	0.1794	64
100	48 h	0.1762	0.0204	0.1360	0.2163	64
100	72 h	0.2262	0.0418	0.1441	0.3084	64
250	6 h	0.1159	0.0084	0.0995	0.1324	64
250	24 h	0.1335	0.0141	0.1058	0.1613	64
250	48 h	0.1308	0.0204	0.0907	0.1710	64
250	72 h	0.1394	0.0418	0.0572	0.2216	64
750	6 h	0.1111	0.0084	0.0946	0.1275	64
750	24 h	0.1115	0.0141	0.0837	0.1393	64
750	48 h	0.1083	0.0204	0.0681	0.1484	64
750	72 h	0.1085	0.0418	0.0263	0.1907	64
MeWo, SRB, alternative						
Concentration [µg/mL]	Time	Mean value	SE	−95% CI	95% CI	N
0	6 h	0.1763	0.0041	0.1682	0.1845	64
0	24 h	0.2738	0.0056	0.2627	0.2849	64
0	48 h	0.4537	0.0122	0.4298	0.4776	64
0	72 h	0.7760	0.0152	0.7460	0.8060	64
10	6 h	0.1748	0.0041	0.1667	0.1830	64
10	24 h	0.2784	0.0056	0.2673	0.2895	64
10	48 h	0.4551	0.0122	0.4312	0.4790	64
10	72 h	0.7916	0.0152	0.7617	0.8216	64
25	6 h	0.1755	0.0041	0.1674	0.1836	64
25	24 h	0.2763	0.0056	0.2652	0.2874	64
25	48 h	0.4471	0.0122	0.4232	0.4710	64
25	72 h	0.7666	0.0152	0.7366	0.7966	64
100	6 h	0.1733	0.0041	0.1652	0.1815	64
100	24 h	0.2797	0.0056	0.2686	0.2908	64
100	48 h	0.4280	0.0122	0.4041	0.4519	64
100	72 h	0.7431	0.0152	0.7131	0.7731	64
250	6 h	0.1759	0.0041	0.1678	0.1840	64
250	24 h	0.2304	0.0056	0.2193	0.2415	64
250	48 h	0.2284	0.0122	0.2045	0.2523	64
250	72 h	0.2809	0.0152	0.2509	0.3109	64
750	6 h	0.1413	0.0041	0.1332	0.1495	64
750	24 h	0.1357	0.0056	0.1246	0.1468	64
750	48 h	0.0800	0.0122	0.0561	0.1039	64
750	72 h	0.0808	0.0152	0.0508	0.1108	64
MeWo, SRB, standard						
Concentration [µg/mL]	TIME	Mean value	SE	−95% CI	95% CI	N
0	6 h	0.1806	0.0072	0.1664	0.1947	64
0	24 h	0.3177	0.0121	0.2940	0.3414	64
0	48 h	0.4323	0.0179	0.3971	0.4675	64
0	72 h	0.9874	0.0389	0.9109	1.0640	64
10	6 h	0.1901	0.0072	0.1759	0.2043	64
10	24 h	0.2821	0.0121	0.2584	0.3058	64
10	48 h	0.4405	0.0179	0.4053	0.4757	64
10	72 h	1.1021	0.0389	1.0255	1.1787	64
25	6 h	0.1880	0.0072	0.1739	0.2022	64
25	24 h	0.2805	0.0121	0.2568	0.3043	64
25	48 h	0.4446	0.0179	0.4094	0.4798	64
25	72 h	0.9703	0.0389	0.8938	1.0469	64
100	6 h	0.1825	0.0072	0.1683	0.1967	64
100	24 h	0.2628	0.0121	0.2391	0.2865	64
100	48 h	0.4324	0.0179	0.3972	0.4676	64
100	72 h	0.9894	0.0389	0.9129	1.0660	64
250	6 h	0.2031	0.0072	0.1889	0.2172	64
250	24 h	0.2391	0.0121	0.2154	0.2629	64
250	48 h	0.2699	0.0179	0.2347	0.3051	64
250	72 h	0.6480	0.0389	0.5714	0.7245	64
750	6 h	0.2128	0.0072	0.1987	0.2270	64
750	24 h	0.2976	0.0121	0.2738	0.3213	64
750	48 h	0.3259	0.0179	0.2907	0.3611	64
750	72 h	0.6160	0.0389	0.5395	0.6926	64
A375, MTT						
Concentration [µg/mL]	Time	Mean value	SE	−95% CI	95% CI	N
0	6 h	0.0877	0.0040	0.0798	0.0956	64
0	24 h	0.1806	0.0062	0.1685	0.1928	64
0	48 h	0.3401	0.0101	0.3203	0.3600	64
0	72 h	0.6923	0.0113	0.6702	0.7145	64
10	6 h	0.0926	0.0040	0.0847	0.1005	64
10	24 h	0.1807	0.0062	0.1686	0.1928	64
10	48 h	0.2420	0.0101	0.2222	0.2619	64
10	72 h	0.2462	0.0113	0.2240	0.2684	64
25	6 h	0.0873	0.0040	0.0794	0.0953	64
25	24 h	0.1635	0.0062	0.1514	0.1756	64
25	48 h	0.2164	0.0101	0.1965	0.2362	64
25	72 h	0.2017	0.0113	0.1795	0.2239	64
100	6 h	0.0796	0.0040	0.0716	0.0875	64
100	24 h	0.1182	0.0062	0.1060	0.1303	64
100	48 h	0.1056	0.0101	0.0857	0.1254	64
100	72 h	0.0934	0.0113	0.0712	0.1156	64
250	6 h	0.0107	0.0040	0.0027	0.0186	64
250	24 h	0.0170	0.0062	0.0049	0.0292	64
250	48 h	0.0117	0.0101	−0.0082	0.0316	64
250	72 h	0.0105	0.0113	−0.0117	0.0327	64
750	6 h	0.0101	0.0040	0.0022	0.0180	64
750	24 h	0.0125	0.0062	0.0004	0.0246	64
750	48 h	0.0093	0.0101	−0.0106	0.0291	64
750	72 h	0.0090	0.0113	−0.0132	0.0312	64
MeWo, MTT						
Concentration [µg/mL]	Time	Mean value	SE	−95% CI	95% CI	N
0	6 h	0.1130	0.0046	0.1039	0.1220	64
0	24 h	0.1697	0.0051	0.1598	0.1797	64
0	48 h	0.2383	0.0067	0.2253	0.2514	64
0	72 h	0.2518	0.0090	0.2342	0.2694	64
10	6 h	0.1176	0.0046	0.1085	0.1266	64
10	24 h	0.1758	0.0051	0.1659	0.1858	64
10	48 h	0.2510	0.0067	0.2379	0.2640	64
10	72 h	0.2783	0.0090	0.2607	0.2959	64
25	6 h	0.1260	0.0046	0.1169	0.1350	64
25	24 h	0.1825	0.0051	0.1726	0.1924	64
25	48 h	0.2551	0.0067	0.2420	0.2681	64
25	72 h	0.2745	0.0090	0.2569	0.2921	64
100	6 h	0.1352	0.0046	0.1262	0.1443	64
100	24 h	0.1974	0.0051	0.1875	0.2074	64
100	48 h	0.2610	0.0067	0.2479	0.2741	64
100	72 h	0.2633	0.0090	0.2457	0.2809	64
250	6 h	0.1099	0.0046	0.1009	0.1190	64
250	24 h	0.1419	0.0051	0.1320	0.1518	64
250	48 h	0.1410	0.0067	0.1279	0.1541	64
250	72 h	0.1035	0.0090	0.0859	0.1212	64
750	6 h	0.0778	0.0046	0.0688	0.0869	64
750	24 h	0.0328	0.0051	0.0229	0.0428	64
750	48 h	0.0163	0.0067	0.0032	0.0294	64
750	72 h	0.0065	0.0090	−0.0111	0.0241	64

**Table A4:** Descriptive statistics of the expected marginal values of absorbance (λ = 520 nm) associated with measurements under the exposition to different type and concentration of *Cornus mas* L extracts (Time*Type*Concentration interaction), measured with use of various assays.

A375, SRB, Alternative							
Type	Concentration [µg/mL]	Time	Mean Value	SE	−95% CI	95% CI	N
yellow	0	6 h	0.1342	0.0045	0.1254	0.142963	32
yellow	0	24 h	0.1801	0.0060	0.1684	0.191846	32
yellow	0	48 h	0.2238	0.0085	0.2070	0.240621	32
yellow	0	72 h	0.2236	0.0086	0.2067	0.240537	32
yellow	10	6 h	0.1315	0.0045	0.1227	0.140329	32
yellow	10	24 h	0.1763	0.0060	0.1645	0.188017	32
yellow	10	48 h	0.1714	0.0085	0.1546	0.188174	32
yellow	10	72 h	0.1393	0.0086	0.1224	0.156243	32
yellow	25	6 h	0.1310	0.0045	0.1222	0.139820	32
yellow	25	24 h	0.1826	0.0060	0.1709	0.194389	32
yellow	25	48 h	0.1628	0.0085	0.1460	0.179618	32
yellow	25	72 h	0.1213	0.0086	0.1044	0.138228	32
yellow	100	6 h	0.1354	0.0045	0.1266	0.144176	32
yellow	100	24 h	0.1569	0.0060	0.1451	0.168617	32
yellow	100	48 h	0.1422	0.0085	0.1254	0.158965	32
yellow	100	72 h	0.1167	0.0086	0.0997	0.133587	32
yellow	250	6 h	0.1014	0.0045	0.0926	0.110167	32
yellow	250	24 h	0.0700	0.0060	0.0582	0.081721	32
yellow	250	48 h	0.0752	0.0085	0.0584	0.092024	32
yellow	250	72 h	0.0900	0.0086	0.0731	0.106928	32
yellow	750	6 h	0.1035	0.0045	0.0947	0.112295	32
yellow	750	24 h	0.0734	0.0060	0.0616	0.085111	32
yellow	750	48 h	0.0737	0.0085	0.0569	0.090518	32
yellow	750	72 h	0.0954	0.0086	0.0785	0.112306	32
red	0	6 h	0.1262	0.0045	0.1174	0.134960	32
red	0	24 h	0.1674	0.0060	0.1557	0.179186	32
red	0	48 h	0.2174	0.0085	0.2006	0.234206	32
red	0	72 h	0.2433	0.0086	0.2264	0.260231	32
red	10	6 h	0.1284	0.0045	0.1196	0.137213	32
red	10	24 h	0.1650	0.0060	0.1532	0.176721	32
red	10	48 h	0.1792	0.0085	0.1624	0.196034	32
red	10	72 h	0.1731	0.0086	0.1562	0.190012	32
red	25	6 h	0.1287	0.0045	0.1199	0.137448	32
red	25	24 h	0.1600	0.0060	0.1482	0.171711	32
red	25	48 h	0.1528	0.0085	0.1360	0.169627	32
red	25	72 h	0.1328	0.0086	0.1159	0.149772	32
red	100	6 h	0.1315	0.0045	0.1227	0.140317	32
red	100	24 h	0.1492	0.0060	0.1375	0.160939	32
red	100	48 h	0.1387	0.0085	0.1219	0.155521	32
red	100	72 h	0.1314	0.0086	0.1144	0.148287	32
red	250	6 h	0.1020	0.0045	0.0932	0.110788	32
red	250	24 h	0.0691	0.0060	0.0573	0.080814	32
red	250	48 h	0.0764	0.0085	0.0596	0.093209	32
red	250	72 h	0.0943	0.0086	0.0774	0.111218	32
red	750	6 h	0.0958	0.0045	0.0870	0.104582	32
red	750	24 h	0.0690	0.0060	0.0573	0.080783	32
red	750	48 h	0.0718	0.0085	0.0550	0.088615	32
red	750	72 h	0.0940	0.0086	0.0771	0.110947	32
A375, SRB, standard							
Type	Concentration [µg/mL]	Time	Mean value	SE	−95% CI	95% CI	N
yellow	0	6 h	0.1116	0.0118	0.0883	0.1349	32
yellow	0	24 h	0.1762	0.0200	0.1369	0.2154	32
yellow	0	48 h	0.3708	0.0289	0.3140	0.4276	32
yellow	0	72 h	1.1145	0.0591	0.9983	1.2307	32
yellow	10	6 h	0.0917	0.0118	0.0684	0.1150	32
yellow	10	24 h	0.1595	0.0200	0.1202	0.1987	32
yellow	10	48 h	0.2422	0.0289	0.1854	0.2990	32
yellow	10	72 h	0.3715	0.0591	0.2553	0.4877	32
yellow	25	6 h	0.1491	0.0118	0.1258	0.1723	32
yellow	25	24 h	0.1868	0.0200	0.1475	0.2260	32
yellow	25	48 h	0.2361	0.0289	0.1794	0.2929	32
yellow	25	72 h	0.3853	0.0591	0.2691	0.5015	32
yellow	100	6 h	0.1473	0.0118	0.1241	0.1706	32
yellow	100	24 h	0.1518	0.0200	0.1125	0.1911	32
yellow	100	48 h	0.1735	0.0289	0.1167	0.2303	32
yellow	100	72 h	0.2186	0.0591	0.1023	0.3348	32
yellow	250	6 h	0.1280	0.0118	0.1047	0.1513	32
yellow	250	24 h	0.1219	0.0200	0.0826	0.1611	32
yellow	250	48 h	0.1376	0.0289	0.0808	0.1944	32
yellow	250	72 h	0.1420	0.0591	0.0258	0.2582	32
yellow	750	6 h	0.1149	0.0118	0.0917	0.1382	32
yellow	750	24 h	0.1250	0.0200	0.0858	0.1643	32
yellow	750	48 h	0.1110	0.0289	0.0543	0.1678	32
yellow	750	72 h	0.1130	0.0591	−0.0032	0.2292	32
red	0	6 h	0.1185	0.0118	0.0952	0.1418	32
red	0	24 h	0.1743	0.0200	0.1350	0.2136	32
red	0	48 h	0.3389	0.0289	0.2822	0.3957	32
red	0	72 h	1.0533	0.0591	0.9371	1.1695	32
red	10	6 h	0.1163	0.0118	0.0930	0.1395	32
red	10	24 h	0.1644	0.0200	0.1252	0.2037	32
red	10	48 h	0.2567	0.0289	0.1999	0.3135	32
red	10	72 h	0.4561	0.0591	0.3399	0.5723	32
red	25	6 h	0.0998	0.0118	0.0765	0.1230	32
red	25	24 h	0.2044	0.0200	0.1651	0.2437	32
red	25	48 h	0.2142	0.0289	0.1574	0.2710	32
red	25	72 h	0.3250	0.0591	0.2088	0.4412	32
red	100	6 h	0.0947	0.0118	0.0714	0.1180	32
red	100	24 h	0.1514	0.0200	0.1121	0.1906	32
red	100	48 h	0.1788	0.0289	0.1221	0.2356	32
red	100	72 h	0.2339	0.0591	0.1177	0.3502	32
red	250	6 h	0.1039	0.0118	0.0806	0.1272	32
red	250	24 h	0.1452	0.0200	0.1059	0.1845	32
red	250	48 h	0.1241	0.0289	0.0673	0.1808	32
red	250	72 h	0.1368	0.0591	0.0206	0.2530	32
red	750	6 h	0.1072	0.0118	0.0839	0.1304	32
red	750	24 h	0.0979	0.0200	0.0587	0.1372	32
red	750	48 h	0.1055	0.0289	0.0488	0.1623	32
red	750	72 h	0.1040	0.0591	−0.0123	0.2202	32
MeWo, SRB, alternative							
Type	Concentration [µg/mL]	Time	Mean value	SE	−95% CI	95% CI	N
yellow	0	6 h	0.1807	0.0058	0.1692	0.1922	32
yellow	0	24 h	0.2772	0.0080	0.2615	0.2929	32
yellow	0	48 h	0.4530	0.0172	0.4192	0.4868	32
yellow	0	72 h	0.7734	0.0216	0.7310	0.8158	32
yellow	10	6 h	0.1811	0.0058	0.1696	0.1926	32
yellow	10	24 h	0.2818	0.0080	0.2661	0.2975	32
yellow	10	48 h	0.4600	0.0172	0.4262	0.4938	32
yellow	10	72 h	0.8032	0.0216	0.7608	0.8457	32
yellow	25	6 h	0.1824	0.0058	0.1709	0.1938	32
yellow	25	24 h	0.2835	0.0080	0.2678	0.2992	32
yellow	25	48 h	0.4641	0.0172	0.4303	0.4979	32
yellow	25	72 h	0.7795	0.0216	0.7371	0.8219	32
yellow	100	6 h	0.1781	0.0058	0.1666	0.1896	32
yellow	100	24 h	0.2897	0.0080	0.2740	0.3054	32
yellow	100	48 h	0.4132	0.0172	0.3794	0.4470	32
yellow	100	72 h	0.7668	0.0216	0.7244	0.8093	32
yellow	250	6 h	0.1795	0.0058	0.1680	0.1910	32
yellow	250	24 h	0.2417	0.0080	0.2261	0.2574	32
yellow	250	48 h	0.2272	0.0172	0.1934	0.2610	32
yellow	250	72 h	0.2917	0.0216	0.2493	0.3341	32
yellow	750	6 h	0.1448	0.0058	0.1333	0.1563	32
yellow	750	24 h	0.1494	0.0080	0.1337	0.1651	32
yellow	750	48 h	0.0915	0.0172	0.0577	0.1253	32
yellow	750	72 h	0.0809	0.0216	0.0385	0.1233	32
red	0	6 h	0.1719	0.0058	0.1605	0.1834	32
red	0	24 h	0.2704	0.0080	0.2547	0.2861	32
red	0	48 h	0.4543	0.0172	0.4205	0.4881	32
red	0	72 h	0.7786	0.0216	0.7362	0.8210	32
red	10	6 h	0.1685	0.0058	0.1570	0.1800	32
red	10	24 h	0.2750	0.0080	0.2593	0.2907	32
red	10	48 h	0.4503	0.0172	0.4165	0.4841	32
red	10	72 h	0.7800	0.0216	0.7376	0.8225	32
red	25	6 h	0.1686	0.0058	0.1571	0.1801	32
red	25	24 h	0.2691	0.0080	0.2534	0.2848	32
red	25	48 h	0.4301	0.0172	0.3963	0.4639	32
red	25	72 h	0.7536	0.0216	0.7112	0.7961	32
red	100	6 h	0.1686	0.0058	0.1571	0.1801	32
red	100	24 h	0.2697	0.0080	0.2540	0.2854	32
red	100	48 h	0.4428	0.0172	0.4090	0.4766	32
red	100	72 h	0.7194	0.0216	0.6770	0.7618	32
red	250	6 h	0.1723	0.0058	0.1608	0.1838	32
red	250	24 h	0.2191	0.0080	0.2034	0.2348	32
red	250	48 h	0.2297	0.0172	0.1959	0.2635	32
red	250	72 h	0.2700	0.0216	0.2276	0.3124	32
red	750	6 h	0.1379	0.0058	0.1264	0.1494	32
red	750	24 h	0.1220	0.0080	0.1063	0.1377	32
red	750	48 h	0.0686	0.0172	0.0348	0.1024	32
red	750	72 h	0.0807	0.0216	0.0383	0.1231	32
MeWo, SRB, standard							
Type	Concentration [µg/mL]	Time	Mean value	SE	−95% CI	95% CI	N
yellow	0	6 h	0.1819	0.0102	0.1619	0.2020	32
yellow	0	24 h	0.3214	0.0171	0.2878	0.3549	32
yellow	0	48 h	0.4323	0.0253	0.3826	0.4821	32
yellow	0	72 h	0.9680	0.0551	0.8597	1.0763	32
yellow	10	6 h	0.2108	0.0102	0.1907	0.2309	32
yellow	10	24 h	0.3100	0.0171	0.2764	0.3435	32
yellow	10	48 h	0.4706	0.0253	0.4209	0.5204	32
yellow	10	72 h	1.1057	0.0551	0.9974	1.2140	32
yellow	25	6 h	0.1982	0.0102	0.1781	0.2182	32
yellow	25	24 h	0.3242	0.0171	0.2907	0.3577	32
yellow	25	48 h	0.4940	0.0253	0.4442	0.5437	32
yellow	25	72 h	1.0551	0.0551	0.9468	1.1633	32
yellow	100	6 h	0.1880	0.0102	0.1679	0.2081	32
yellow	100	24 h	0.2827	0.0171	0.2492	0.3163	32
yellow	100	48 h	0.4865	0.0253	0.4367	0.5362	32
yellow	100	72 h	1.0251	0.0551	0.9168	1.1334	32
yellow	250	6 h	0.2097	0.0102	0.1896	0.2297	32
yellow	250	24 h	0.2765	0.0171	0.2430	0.3101	32
yellow	250	48 h	0.3123	0.0253	0.2625	0.3620	32
yellow	250	72 h	0.7511	0.0551	0.6428	0.8594	32
yellow	750	6 h	0.2079	0.0102	0.1878	0.2279	32
yellow	750	24 h	0.3127	0.0171	0.2791	0.3462	32
yellow	750	48 h	0.4503	0.0253	0.4006	0.5001	32
yellow	750	72 h	0.6667	0.0551	0.5584	0.7750	32
red	0	6 h	0.1792	0.0102	0.1592	0.1993	32
red	0	24 h	0.3141	0.0171	0.2805	0.3476	32
red	0	48 h	0.4323	0.0253	0.3825	0.4820	32
red	0	72 h	1.0069	0.0551	0.8986	1.1152	32
red	10	6 h	0.1694	0.0102	0.1494	0.1895	32
red	10	24 h	0.2542	0.0171	0.2206	0.2877	32
red	10	48 h	0.4104	0.0253	0.3606	0.4602	32
red	10	72 h	1.0985	0.0551	0.9902	1.2068	32
red	25	6 h	0.1779	0.0102	0.1578	0.1979	32
red	25	24 h	0.2369	0.0171	0.2033	0.2704	32
red	25	48 h	0.3953	0.0253	0.3455	0.4451	32
red	25	72 h	0.8856	0.0551	0.7773	0.9939	32
red	100	6 h	0.1771	0.0102	0.1570	0.1971	32
red	100	24 h	0.2429	0.0171	0.2094	0.2764	32
red	100	48 h	0.3783	0.0253	0.3285	0.4281	32
red	100	72 h	0.9538	0.0551	0.8455	1.0621	32
red	250	6 h	0.1964	0.0102	0.1764	0.2165	32
red	250	24 h	0.2018	0.0171	0.1682	0.2353	32
red	250	48 h	0.2275	0.0253	0.1777	0.2772	32
red	250	72 h	0.5448	0.0551	0.4365	0.6531	32
red	750	6 h	0.2178	0.0102	0.1977	0.2379	32
red	750	24 h	0.2825	0.0171	0.2489	0.3160	32
red	750	48 h	0.2014	0.0253	0.1516	0.2512	32
red	750	72 h	0.5654	0.0551	0.4571	0.6736	32
A375, MTT							
Type	Concentration [µg/mL]	Time	Mean value	SE	−95% CI	95% CI	N
yellow	0	6 h	0.0869	0.0057	0.0757	0.0981	32
yellow	0	24 h	0.1877	0.0087	0.1706	0.2049	32
yellow	0	48 h	0.3512	0.0143	0.3231	0.3793	32
yellow	0	72 h	0.7270	0.0159	0.6956	0.7584	32
yellow	10	6 h	0.0977	0.0057	0.0865	0.1089	32
yellow	10	24 h	0.1977	0.0087	0.1806	0.2149	32
yellow	10	48 h	0.2257	0.0143	0.1976	0.2537	32
yellow	10	72 h	0.1910	0.0159	0.1596	0.2224	32
yellow	25	6 h	0.0980	0.0057	0.0867	0.1092	32
yellow	25	24 h	0.1760	0.0087	0.1589	0.1932	32
yellow	25	48 h	0.2246	0.0143	0.1966	0.2527	32
yellow	25	72 h	0.2110	0.0159	0.1796	0.2423	32
yellow	100	6 h	0.0890	0.0057	0.0778	0.1002	32
yellow	100	24 h	0.1162	0.0087	0.0990	0.1333	32
yellow	100	48 h	0.0942	0.0143	0.0661	0.1223	32
yellow	100	72 h	0.0749	0.0159	0.0435	0.1063	32
yellow	250	6 h	0.0089	0.0057	−0.0023	0.0201	32
yellow	250	24 h	0.0169	0.0087	−0.0002	0.0341	32
yellow	250	48 h	0.0132	0.0143	−0.0149	0.0413	32
yellow	250	72 h	0.0068	0.0159	−0.0246	0.0382	32
yellow	750	6 h	0.0046	0.0057	−0.0066	0.0158	32
yellow	750	24 h	0.0136	0.0087	−0.0036	0.0307	32
yellow	750	48 h	0.0090	0.0143	−0.0191	0.0371	32
yellow	750	72 h	0.0093	0.0159	−0.0220	0.0407	32
red	0	6 h	0.0885	0.0057	0.0773	0.0997	32
red	0	24 h	0.1735	0.0087	0.1564	0.1907	32
red	0	48 h	0.3291	0.0143	0.3010	0.3572	32
red	0	72 h	0.6577	0.0159	0.6263	0.6891	32
red	10	6 h	0.0875	0.0057	0.0763	0.0987	32
red	10	24 h	0.1637	0.0087	0.1465	0.1808	32
red	10	48 h	0.2584	0.0143	0.2303	0.2865	32
red	10	72 h	0.3014	0.0159	0.2700	0.3328	32
red	25	6 h	0.0767	0.0057	0.0655	0.0880	32
red	25	24 h	0.1510	0.0087	0.1338	0.1682	32
red	25	48 h	0.2081	0.0143	0.1800	0.2362	32
red	25	72 h	0.1924	0.0159	0.1610	0.2238	32
red	100	6 h	0.0701	0.0057	0.0589	0.0813	32
red	100	24 h	0.1202	0.0087	0.1030	0.1373	32
red	100	48 h	0.1169	0.0143	0.0888	0.1450	32
red	100	72 h	0.1119	0.0159	0.0806	0.1433	32
red	250	6 h	0.0124	0.0057	0.0012	0.0236	32
red	250	24 h	0.0171	0.0087	0.0000	0.0343	32
red	250	48 h	0.0102	0.0143	−0.0179	0.0382	32
red	250	72 h	0.0142	0.0159	−0.0171	0.0456	32
red	750	6 h	0.0156	0.0057	0.0044	0.0268	32
red	750	24 h	0.0114	0.0087	−0.0057	0.0286	32
red	750	48 h	0.0095	0.0143	−0.0186	0.0376	32
red	750	72 h	0.0087	0.0159	−0.0227	0.0400	32
MeWo, MTT							
Type	Concentration [µg/mL]	Time	Mean value	SE	−95% CI	95% CI	N
yellow	0	6 h	0.1103	0.0065	0.0975	0.1230	32
yellow	0	24 h	0.1826	0.0071	0.1686	0.1967	32
yellow	0	48 h	0.2484	0.0094	0.2299	0.2669	32
yellow	0	72 h	0.2711	0.0127	0.2462	0.2960	32
yellow	10	6 h	0.1181	0.0065	0.1053	0.1309	32
yellow	10	24 h	0.1805	0.0071	0.1664	0.1945	32
yellow	10	48 h	0.2626	0.0094	0.2441	0.2811	32
yellow	10	72 h	0.2922	0.0127	0.2673	0.3171	32
yellow	25	6 h	0.1296	0.0065	0.1169	0.1424	32
yellow	25	24 h	0.1867	0.0071	0.1726	0.2007	32
yellow	25	48 h	0.2748	0.0094	0.2563	0.2933	32
yellow	25	72 h	0.2795	0.0127	0.2546	0.3045	32
yellow	100	6 h	0.1407	0.0065	0.1279	0.1535	32
yellow	100	24 h	0.1966	0.0071	0.1826	0.2107	32
yellow	100	48 h	0.2659	0.0094	0.2474	0.2844	32
yellow	100	72 h	0.2548	0.0127	0.2299	0.2797	32
yellow	250	6 h	0.1174	0.0065	0.1046	0.1302	32
yellow	250	24 h	0.1495	0.0071	0.1355	0.1636	32
yellow	250	48 h	0.1674	0.0094	0.1489	0.1859	32
yellow	250	72 h	0.1081	0.0127	0.0832	0.1330	32
yellow	750	6 h	0.0925	0.0065	0.0797	0.1053	32
yellow	750	24 h	0.0462	0.0071	0.0321	0.0602	32
yellow	750	48 h	0.0183	0.0094	−0.0002	0.0368	32
yellow	750	72 h	0.0065	0.0127	−0.0184	0.0314	32
red	0	6 h	0.1157	0.0065	0.1029	0.1285	32
red	0	24 h	0.1569	0.0071	0.1428	0.1709	32
red	0	48 h	0.2283	0.0094	0.2098	0.2468	32
red	0	72 h	0.2325	0.0127	0.2076	0.2574	32
red	10	6 h	0.1170	0.0065	0.1042	0.1298	32
red	10	24 h	0.1712	0.0071	0.1571	0.1852	32
red	10	48 h	0.2394	0.0094	0.2209	0.2579	32
red	10	72 h	0.2643	0.0127	0.2394	0.2892	32
red	25	6 h	0.1223	0.0065	0.1095	0.1351	32
red	25	24 h	0.1783	0.0071	0.1643	0.1923	32
red	25	48 h	0.2353	0.0094	0.2168	0.2538	32
red	25	72 h	0.2695	0.0127	0.2445	0.2944	32
red	100	6 h	0.1298	0.0065	0.1170	0.1426	32
red	100	24 h	0.1982	0.0071	0.1842	0.2123	32
red	100	48 h	0.2561	0.0094	0.2376	0.2746	32
red	100	72 h	0.2718	0.0127	0.2469	0.2967	32
red	250	6 h	0.1024	0.0065	0.0896	0.1152	32
red	250	24 h	0.1343	0.0071	0.1202	0.1483	32
red	250	48 h	0.1146	0.0094	0.0961	0.1331	32
red	250	72 h	0.0990	0.0127	0.0741	0.1239	32
red	750	6 h	0.0631	0.0065	0.0503	0.0759	32
red	750	24 h	0.0194	0.0071	0.0054	0.0335	32
red	750	48 h	0.0143	0.0094	−0.0042	0.0328	32
red	750	72 h	0.0065	0.0127	−0.0184	0.0314	32

## Appendix B

**Table A5:** Logistic regression models used to estimate the IC_50_ values associated with the observed cytotoxic effect of *Cornus mas* L. extracts on selected melanoma cell lines (A375, MeWo).

Cell Line	Method	Time	Viability Equation (where: Y—Cytotoxic Response (% Viability); X—Concentration of *C. mas* L. Extract)	Calculated IC_50_ [µg/mL]
A375	MTT	6 h	$Y=\frac{102.6122}{{\left(1+\frac{x}{188.6701}\right)}^{3.1319\;}}$	188.6701
A375	MTT	24 h	$Y=\frac{101.5023}{{\left(1+\frac{x}{138.4745}\right)}^{1.5585\;}}$	138.4745
A375	MTT	48 h	$Y=\frac{99.6791}{{\left(1+\frac{x}{58.8851\;}\right)}^{0.9029}}$	58.8851
A375	MTT	72 h	$Y=\frac{100.0238}{{\left(1+\frac{x}{9.9146}\right)}^{0.432}}$	9.9146
A375	SRB (alternative)	6 h	$Y=\frac{102.0205}{{\left(1+\frac{x}{2611.8321}\right)}^{0.7514}}$	2611.8321
A375	SRB (alternative)	24 h	$Y=\frac{103.5300}{{\left(1+\frac{x}{338.5524\;}\right)}^{0.8981}}$	338.5524
A375	SRB (alternative)	48 h	$Y=\frac{100.018}{{\left(1+\frac{x}{182.7961}\right)}^{0.5007}}$	182.7961
A375	SRB (alternative)	72 h	$Y=\frac{100.1688}{{\left(1+\frac{x}{205.9856}\right)}^{0.2361\;}}$	205.9856
A375	SRB (standard)	6 h	-	Non-computable
A375	SRB (standard)	24 h	$Y=\frac{103.2968}{{\left(1+\frac{x}{3548.8126}\right)}^{0.6808}}$	3548.8126
A375	SRB (standard)	48 h	$Y=\frac{100.0344}{{\left(1+\frac{x}{339.5497\;}\right)}^{0.3113\;}}$	339.5497
A375	SRB (standard)	72 h	$Y=\frac{100.0213}{{\left(1+\frac{x}{6.4458}\right)}^{0.2956}}$	6.4458
MeWo	MTT	6 h	$Y=\frac{110.6273}{{\left(1+\frac{x}{970.1337}\right)}^{1.9727\;}}$	970.1337
MeWo	MTT	24 h	$Y=\frac{107.4500}{{\left(1+\frac{x}{416.2932}\right)}^{2.816}}$	416.2932
MeWo	MTT	48 h	$Y=\frac{106.1392}{{\left(1+\frac{x}{265.4668\;}\right)}^{4.9316\;}}$	265.4668
MeWo	MTT	72 h	$Y=\frac{107.0591}{{\left(1+\frac{x}{232.6805}\right)}^{5.1644\;}}$	232.6805
MeWo	SRB (alternative)	6 h	$Y=\frac{99.5526}{{\left(1+\frac{x}{897.7824}\right)}^{8.2243}}$	897.7824
MeWo	SRB (alternative)	24 h	$Y=\frac{101.8792}{{\left(1+\frac{x}{727.0854}\right)}^{1.6182\;}}$	727.0854
MeWo	SRB (alternative)	48 h	$Y=\frac{106.1392}{{\left(1+\frac{x}{265.4668}\right)}^{4.9316}}$	265.4668
MeWo	SRB (alternative)	72 h	$Y=\frac{100.5127}{{\left(1+\frac{x}{276.0806}\right)}^{1.8460}}$	276.0806
MeWo	SRB (standard)	6 h	-	Non-computable
MeWo	SRB (standard)	24 h	$Y=\frac{90.9666}{{\left(1+\frac{x}{2317.357}\right)}^{8.6443}}$	2317.357
MeWo	SRB (standard)	48 h	$Y=\frac{104.6794}{{\left(1+\frac{x}{2190.8609}\right)}^{0.6040}}$	2190.8609
MeWo	SRB (standard)	72 h	$Y=\frac{106.4493}{{\left(1+\frac{x}{920.6867}\right)}^{0.8051}}$	920.6867

The terms ‘standard’ and ‘alternative’ refer to different assay protocols used for SRB assay, described in the ‘Methods’ section. ‘Non-computable’ in context of the IC_50_ values was imputed when no relative cell viability decrease was observed for a given time of cell culture.
