# Supplementary material for: Cornelian Cherry (Cornus mas L.) Extracts Exert Cytotoxicity in Two Selected Melanoma Cell Lines—A Factorial Analysis of Time-Dependent Alterations in Values Obtained with SRB and MTT Assays
Source: Molecules. 2022 Jun 29;27(13):4193. doi: 10.3390/molecules27134193 (PMC9268180; doi:10.3390/molecules27134193)
Supplement: Supplementary file 1 [file molecules-27-04193-s001.zip › Table_S1.pdf]

Table S1. Identification and content (mg/100 g dry weight (dw)) of main compounds of extracts from yellow and red Cornelian cherry (*Cornus mas* L.) fruits by means of LC-MS and HPLC.

Compound	UV λmax (nm)	Ionisation mode	MW [Da]	MS[1] [M – H]⁻/ [M – H]⁺ (*m/z*)	MS[2] fragment ions (*m/z*)	Yellow *C. mas* extract (mg/100 g dw)	Red *C. mas* extract (mg/100 g dw)
				**IRIDOIDS**			
Loganic acid	245	-	376	375	213	15383.35±8.5	15432.33±29.48
Cornuside	245/273	-	542	541	169	1538.97±5.77	1169.28±17.19
**Total iridoids**						**16922.32**	**16601.62**
				**ANTHOCYANINS**			
Cyanidin 3-*O*-galactoside	515	+	449^+	449	287	0.00	768.37±11.40
Cyanidin 3-*O*-robinobioside	516	+	595^+	595	287	0.00	197.67±3.18
Pelargonidin 3-*O*-galactoside	501	+	433^+	433	271	0.00	1101.65±13.21
Pelargonidin 3-*O*-robinobioside	501	+	579^+	579	271	0.00	133.80±3.56
**Total anthocyanins**						**0.00**	**2201.49**
				**PHENOLIC ACIDS**			
trans-Caftaric acid	324	-	312	311	179/149	283.63±0.0	296.59±0.0
Caftaric acid isomer	324	-	312	311	179/149	584.34±12.19	308.51±1.81
Coutaric acid isomer	311	-	296	295	163/149	187.59±0.56	92,63±3.88
**Total phenolic acids**						**1055.56**	**697.73**
				**FLAVONOLS**			
Quercetin 3-*O*-glucuronide	354	-	478	477	301	196.48±4.47	139.93±5.93
Kaempferol 3-*O*-galactoside	348	-	448	447	285	0.00	100.90±4.81
**Total flavonols**						**196.48**	**240.83**
				**HYDROLYSABLE TANNINS**			
Total gemin D isomers	265	-	634	633	301/275/249/169	228.84	903.33
Tellimagrandin I isomers	267	-	786	785	301/275/249/169	2510.66	276.11
Tellimagrandin II	271	-	938	937	785/633/301/275/249/169	0.00	595.47
Total camptothin A isomers	264	-	1418	708^{-2}, 1417	1247/783/633/301	2943.69	2294.22
Total cornusiin A isomers	273	-	1570	784^{-2}, 1569	935/633/313/301	2875.13	8503.24
Total cornusiin F isomers	277	-	2202	1100^{-2}, 2201	1569/785/633/301	1063.77	0.00
Total cornusiin C isomers	268	-	2354	1176^{-2}, 2353	786/633/451/301	3222.09	4249.05
**Total hydrolysable tannins**						**18722.01**	**21686.80**
