# Supplementary figures and images for: Cornelian Cherry (Cornus mas L.) Extracts Exert Cytotoxicity in Two Selected Melanoma Cell Lines—A Factorial Analysis of Time-Dependent Alterations in Values Obtained with SRB and MTT Assays
Source: Molecules. 2022 Jun 29;27(13):4193. doi: 10.3390/molecules27134193 (PMC9268180; doi:10.3390/molecules27134193)

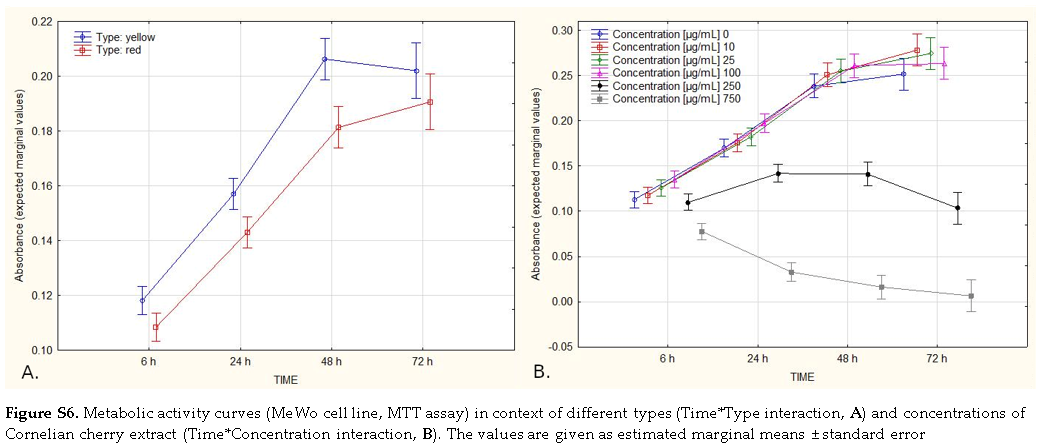

Supplement: Supplementary file 1 [file molecules-27-04193-s001.zip › Fig_S6.png]

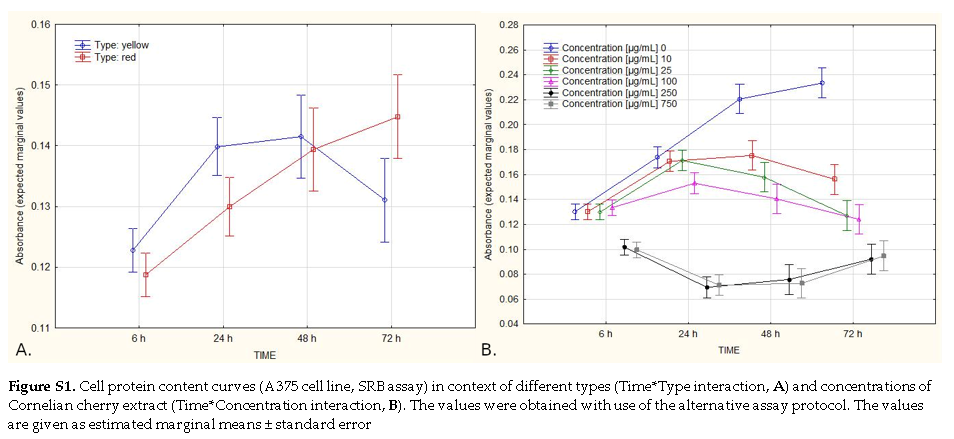

Supplement: Supplementary file 1 [file molecules-27-04193-s001.zip › Fig_S1.png]

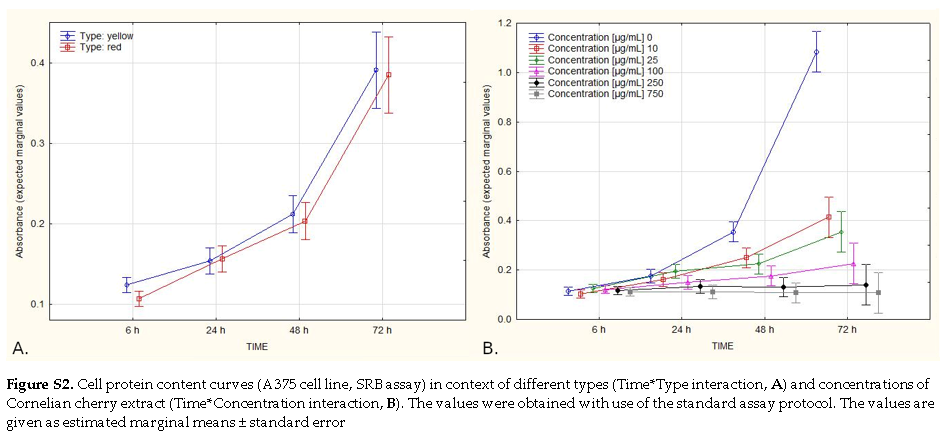

Supplement: Supplementary file 1 [file molecules-27-04193-s001.zip › Fig_S2.png]

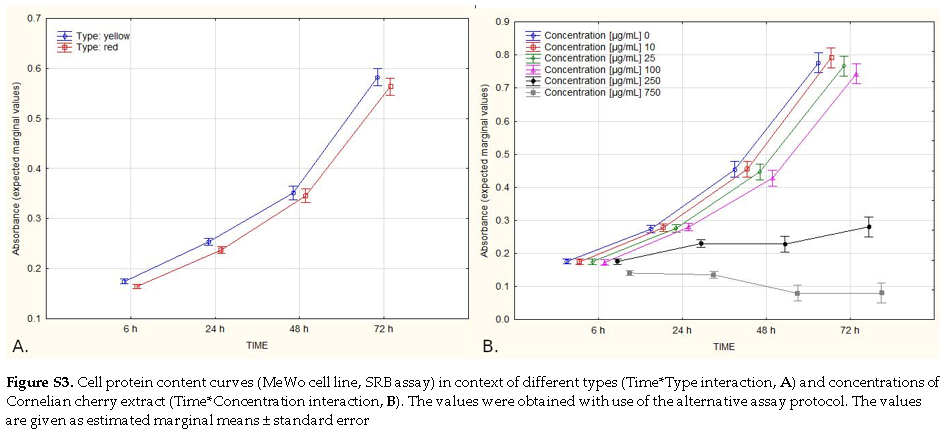

Supplement: Supplementary file 1 [file molecules-27-04193-s001.zip › Fig_S3.png]

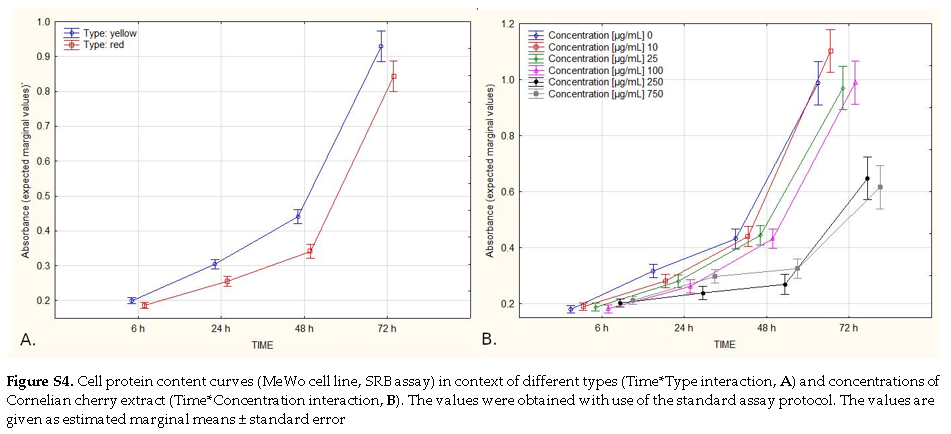

Supplement: Supplementary file 1 [file molecules-27-04193-s001.zip › Fig_S4.png]

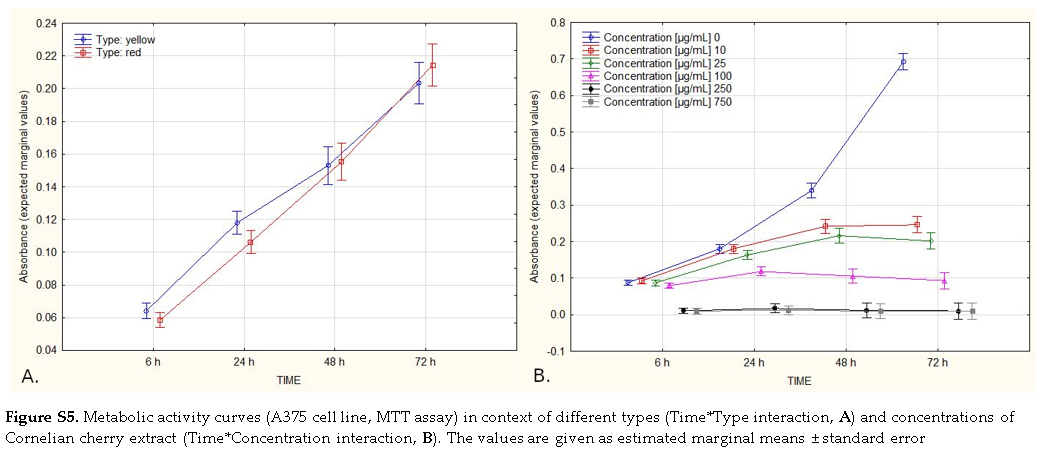

Supplement: Supplementary file 1 [file molecules-27-04193-s001.zip › Fig_S5.png]
